# TeaNeRF: an integrated 3D visual perception pipeline for tea bud harvesting

**DOI:** 10.3389/fpls.2026.1739203

**Published:** 2026-03-02

**Authors:** Weiheng Chen, Xun Li, Lei Rao, Xiang Xia

**Affiliations:** 1 School of Computer Science and Engineering, Wuhan Institute of Technology, Wuhan, China; 2 Hubei Key Laboratory of Intelligent Robot, Wuhan Institute of Technology, Wuhan, China

**Keywords:** 3D reconstruction, automated harvesting, NeRF, SAM2, semantic segmentation, tea bud detection, YOLOv11

## Abstract

Accurate perception of tea buds is a fundamental prerequisite for intelligent and precise tea harvesting planning. However, in real tea plantation environments, reliable harvesting-oriented perception at the planning level remains highly challenging due to the small size of tea buds, severe occlusion, complex background clutter, and the lack of accurate three-dimensional spatial information. To address these challenges, we propose TeaNeRF, an integrated three-dimensional visual perception pipeline designed for harvesting-oriented tea bud analysis. Instead of treating detection, segmentation, and spatial analysis as independent tasks, TeaNeRF integrates sequential two-dimensional recognition, monocular depth estimation, and neural radiance field reconstruction into a coherent perception pipeline, allowing accurate spatial understanding of tea buds in complex natural scenes. It should be noted that the proposed integration is conducted at the perception-output level, where multiple modular components are connected through fixed interfaces, rather than through joint optimization or an end-to-end trainable formulation. The proposed framework combines an enhanced YOLO-based detector, prompt-guided segmentation, and monocular depth priors to guide NeRF-based three-dimensional reconstruction. By incorporating depth supervision and semantic-aware neural fields, TeaNeRF generates dense and geometrically consistent point clouds with reliable semantic separation. Quantitative evaluations show consistent improvements in reconstruction fidelity, as reflected by increased PSNR and reduced LPIPS across multiple tea tree scenes. Based on the reconstructed semantic point cloud, a three-dimensional clustering and geometric fitting strategy is further developed to enable tea bud counting and harvesting-oriented candidate point estimation at the perception level. Experiments conducted on a real-world dataset of 4,700 tea plantation images demonstrate that TeaNeRF improves detection accuracy (mAP@50 = 91.7%), segmentation quality (IoU = 0.640), and overall three-dimensional perception performance. Case-level counting results on representative tea trees indicate that the proposed 3D semantic point cloud–based approach can provide feasible tea bud counting behavior and consistent spatial guidance cues for downstream harvesting planning. By providing structured three-dimensional spatial information, including tea bud locations, counts, and harvesting-oriented candidate points, TeaNeRF offers practical perception-level outputs for downstream planning in automated tea harvesting systems.

## 1 Introduction

Tea is a major agricultural product in China, supported by a long-standing tea-drinking tradition and a rapidly expanding market. China accounts for approximately 50% of global tea production, ranking as the world’s largest producer (Yu and He, 2018). Tea harvesting is highly seasonal and labor-intensive, creating substantial workforce demand during peak periods. Although reciprocating cutting tea plucking machines and adaptive canopy-following control strategies have been developed to improve mechanized harvesting stability (Zhang et al., 2025), they often damage tea buds and unintentionally collect mature leaves, limiting product quality. As a result, manual picking remains the dominant harvesting method. These limitations indicate that precise and selective tea bud harvesting is not merely a mechanical challenge, but fundamentally a perception problem. Harvesting-oriented automation requires accurate tea bud identification and reliable three-dimensional spatial understanding in complex plantation environments, which are not sufficiently supported by existing harvesting systems.

To realize the mechanization and precision of tea picking, accurate identification and localization of tea buds in complex but controlled field environments are essential. Early studies relied primarily on traditional image processing techniques for tea shoot detection. For example, Yang et al. (Yang et al., 2009) achieved a recognition accuracy of 94% using color component extraction and edge detection. Wu et al. (Wu et al., 2013) analyzed the color differences between the tea buds and the background regions based on components G and B, reporting recognition accuracies that exceed 92%. Long et al. (Long et al., 2022) proposed an image segmentation approach based on ultra-green features and the Otsu thresholding method, combined with morphological operations, to effectively extract tea shoot regions. Despite these promising results under relatively controlled conditions, traditional image processing methods heavily depend on hand-crafted features and predefined thresholds, making them highly sensitive to illumination variation, background clutter, and growth-stage diversity in real tea plantation environments. As a result, their robustness and generalization ability are limited in practical harvesting scenarios, motivating the adoption of learning-based approaches with stronger feature representation capability.

With the rapid development of deep learning, tea bud perception has increasingly adopted neural network–based methods to overcome the limitations of hand-crafted features, particularly for small-target detection in complex but controlled field environments. Compared with traditional image processing approaches, deep models learn hierarchical feature representations with improved robustness and generalization. Representative studies have focused on enhancing YOLO-based detectors through multi-scale feature extraction, lightweight architectures, and improved loss designs. Zhou et al. (2024) proposed an optimized YOLOv8-based framework for tea bud detection and yield estimation, demonstrating the feasibility of integrating object detection with production-related analysis in tea plantation environments. For example, Yang et al. (Yang et al., 2019) and Wang et al. (Wang et al., 2024, Wang et al., 2025) improved detection performance by introducing pyramid structures, lightweight convolutions, and decoupled heads, while Gui et al. (Gui et al., 2023, Gui et al., 2024), Liu et al. (Liu et al., 2024), and Jianqiang et al. (Jianqiang et al., 2024) emphasized lightweight design and robustness under occlusion and high background similarity. Beyond detection, several studies explored two-dimensional segmentation and picking point localization, including attention-enhanced semantic segmentation networks (Zhang et al., 2023a; Chen et al., 2024a) and joint detection–keypoint frameworks based on heatmap supervision or instance segmentation (Song et al., 2025; Yan et al., 2022). Pan et al. (Pan et al., 2024) further combined traditional algorithms with Transformer-based detection and segmentation models to improve performance across multiple tea categories. Zhu et al. (Zhu et al., 2023a) proposed a tea bud detection and localization method based on an improved YOLOv5s model combined with 3D point cloud processing, enabling three-dimensional picking point estimation from detected regions. Despite these advances, existing deep learning–based methods predominantly operate in two-dimensional image space and remain limited in providing reliable harvesting-oriented spatial information. Severe occlusion, bud–leaf adhesion, and depth ambiguity often lead to inaccurate localization when inferring picking points from 2D results alone. Consequently, although deep models substantially improve detection and segmentation accuracy, they still fall short of delivering explicit three-dimensional spatial cues required for precise and automated tea bud harvesting.

In recent years, three-dimensional reconstruction technologies have been increasingly adopted in agricultural and crop-related studies, as they provide richer and more accurate spatial information than conventional two-dimensional methods and enable multi-view structural analysis of plants. Representative works have combined deep learning–based detection with point cloud reconstruction to estimate picking points (Zhu et al., 2023b), employed UAV-based multi-view imagery for crop canopy reconstruction (Zhu et al., 2023), and developed multi-sensor robotic platforms for high-resolution 3D plant scanning in field environments (Esser et al., 2023). Three-dimensional reconstruction has also been widely explored for agricultural phenotyping, including NeRF-based peanut plant reconstruction (Saeed et al., 2023), 3D Gaussian splatting for cotton phenotyping (Jiang et al., 2024), and SfM-MVS–based beet reconstruction for trait extraction (Chen et al., 2024b). More recently, NeRF has been applied to complex orchard and crop scenes, such as strawberry garden reconstruction (Zhang et al., 2024), rice spike reconstruction combining YOLOv8 and SAM (Yang et al., 2024b), and fruit yield estimation using FruitNeRF (Meyer et al., 2024). From a harvesting-oriented perspective, however, most existing 3D reconstruction and NeRF-based agricultural studies primarily focus on phenotyping, yield estimation, or visual rendering quality, rather than actionable harvesting perception. In particular, they often lack tight integration with robust two-dimensional detection and segmentation, fail to produce dense and reliable bud-level semantic point clouds, and do not explicitly generate precise three-dimensional cues, such as picking points, required for automated harvesting. These limitations motivate the development of an integrated 3D perception pipeline that bridges two-dimensional recognition and three-dimensional reconstruction for practical tea bud harvesting.

In summary, although significant progress has been made in tea bud detection, segmentation, and three-dimensional crop reconstruction, existing approaches remain insufficient to meet the practical requirements of automated tea harvesting. Current methods either lack robustness in complex natural environments, fail to provide dense and reliable bud-level spatial representations, or do not effectively integrate two-dimensional perception with high-fidelity three-dimensional reconstruction.

To address these challenges, this study proposes TeaNeRF, an integrated three-dimensional visual perception pipeline tailored for tea bud harvesting planning. By coherently integrating robust 2D detection and segmentation with depth-aware neural radiance field reconstruction, TeaNeRF enables accurate three-dimensional localization, counting, and candidate picking point estimation of tea buds in complex but controlled field environments, thereby providing a practical and reliable perception-level foundation for mechanized tea harvesting preparation.

The main contributions of this study are summarized as follows:

1. We formulate tea bud harvesting as an integrated three-dimensional visual perception problem and propose a harvesting-oriented perception pipeline that bridges two-dimensional recognition and three-dimensional reconstruction.
2. We develop a depth-enhanced semantic NeRF representation that supports bud-level three-dimensional reconstruction and semantic point cloud generation for individual tea plants under real-world imaging conditions.
3. Based on the reconstructed semantic point cloud, we enable accurate tea bud counting and three- dimensional harvesting-oriented candidate point estimation, providing actionable spatial cues for automated tea harvesting.

## 2 Materials and methods

### 2.1 Pipeline overview

**Figure 1:**
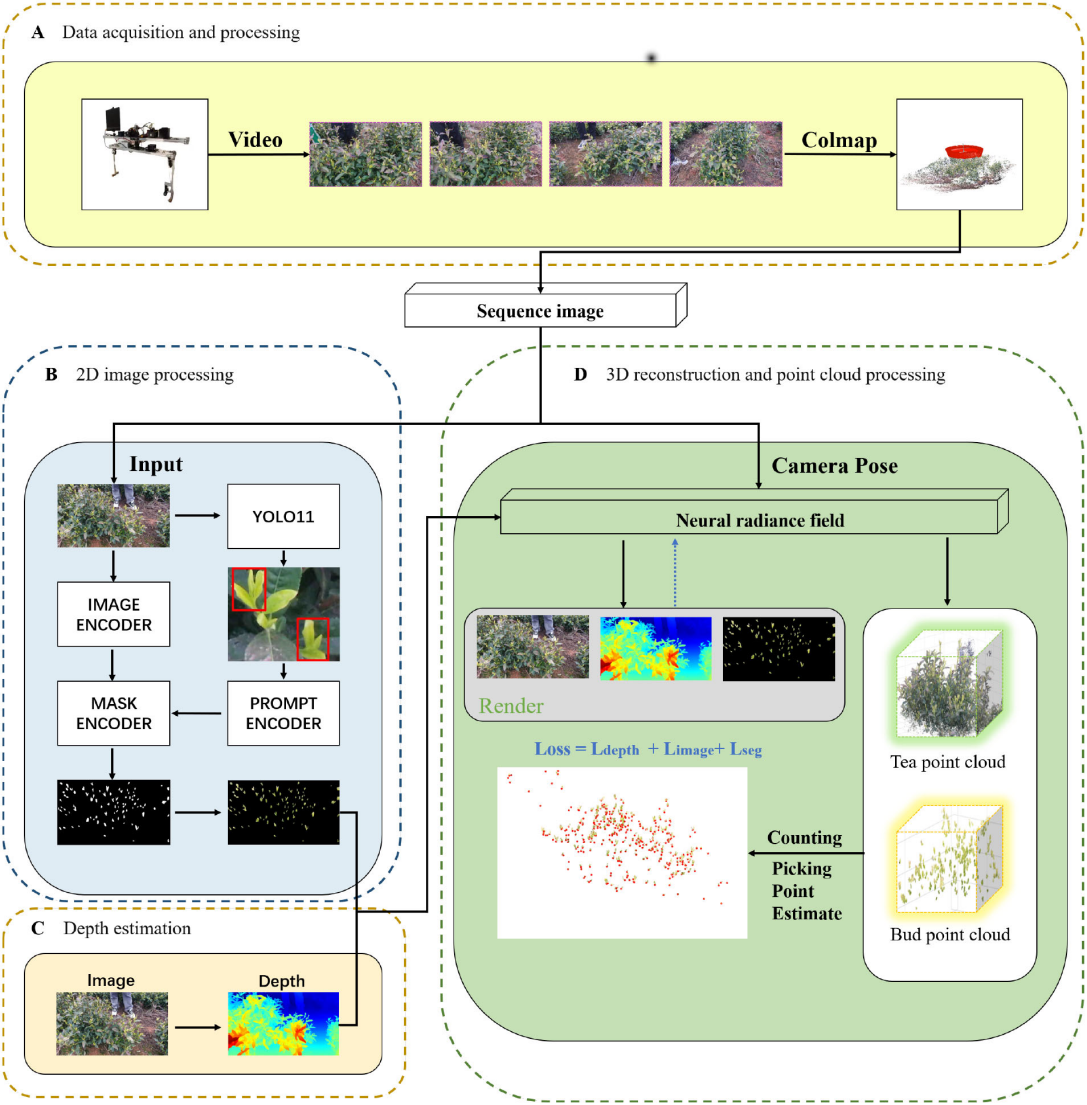
Overview of the proposed TeaNeRF pipeline. **(A)** Multi-view images of tea trees are acquired, and camera poses are estimated using COLMAP; **(B)** YOLOv11 is employed to detect tea buds, and the detection results are used to guide SAM2 for accurate semantic segmentation; **(C)** Depth Anything v2 is applied to estimate monocular depth maps; **(D)** Based on the images and depth priors, a semantic NeRF model reconstructs the 3D structure of the tea tree, from which bud-level point clouds are extracted for tea bud counting and harvesting-oriented candidate point localization.

In this paper, we propose an integrated three-dimensional visual perception pipeline that integrates tea bud recognition, semantic segmentation, counting, and harvesting-oriented candidate picking point estimation to support harvesting planning and preparation in real tea plantation environments. The overall workflow of the proposed framework is illustrated in **Figure 1**.

The selection of YOLOv11, SAM2, Depth Anything V2, and NeRF was guided by a balance between accuracy, robustness, and practical applicability in complex outdoor harvesting environments. YOLOv11 provides strong performance for small-object detection with high efficiency, while SAM2 enables flexible and accurate segmentation with minimal annotation overhead. Depth Anything V2 offers reliable monocular depth estimation with strong cross-scene generalization, which is well-suited for natural environments. NeRF, combined with depth supervision and semantic rendering, is adopted to obtain spatially coherent fine-scale geometry under repetitive textures and self-occlusion, providing a coherent three-dimensional representation that better supports subsequent harvesting-oriented analysis.

### 2.2 Data acquisition

This study focuses on tea bud image acquisition in complex outdoor environments, and all data were collected autonomously. To improve acquisition efficiency and satisfy the requirements of subsequent 3D reconstruction, an automated image acquisition system was developed. The device enables controlled rotation around individual tea trees and flexible adjustment of the number of captured images by setting the acquisition interval.

**Figure 2:**
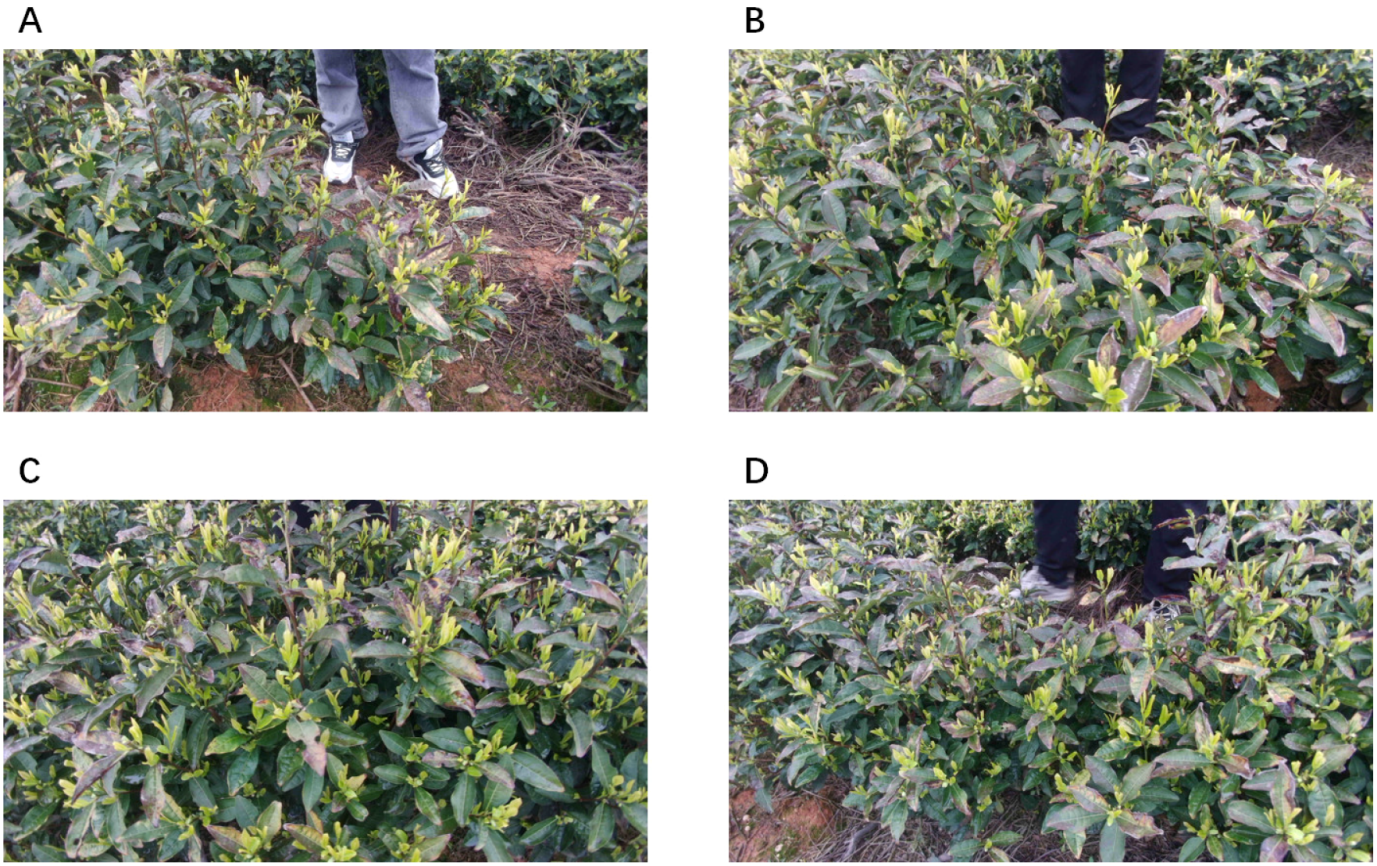
Tea tree images captured from different viewpoints. **(A–D)** Representative images of the same tea tree captured from four different perspectives.

All images were captured using an Obsmeet 4K camera, with a resolution of 3840 × 2160 pixels and stored in JPG format. Data collection was conducted in April under natural field conditions in Chongyang County, Xianning City, Hubei Province, China. The final dataset contains 4,700 static images of yellow tea buds, covering diverse viewpoints and occlusion conditions, as illustrated in **Figure 2**. Tea bud targets in the images were manually annotated using the Trex annotation tool to generate bounding-box labels for model training.

The dataset used in this study was collected from a single geographic region and tea variety, which limits direct evaluation of cross-region and cross-variety generalization. The primary objective of this work is to validate the feasibility and effectiveness of a harvesting-oriented three-dimensional perception pipeline under controlled field conditions, rather than to establish generalization across different tea cultivars, growth stages, or environmental settings. Systematic evaluation under more diverse regions, tea varieties, and growth conditions will be explored in future work.

### 2.3 Data preprocessing

In the data preprocessing stage, COLMAP was first employed to estimate the camera poses (including position and orientation) of the original images, thereby restoring the geometric information during image acquisition; in this work, COLMAP is used for camera pose estimation rather than dense surface reconstruction.

**Figure 3:**
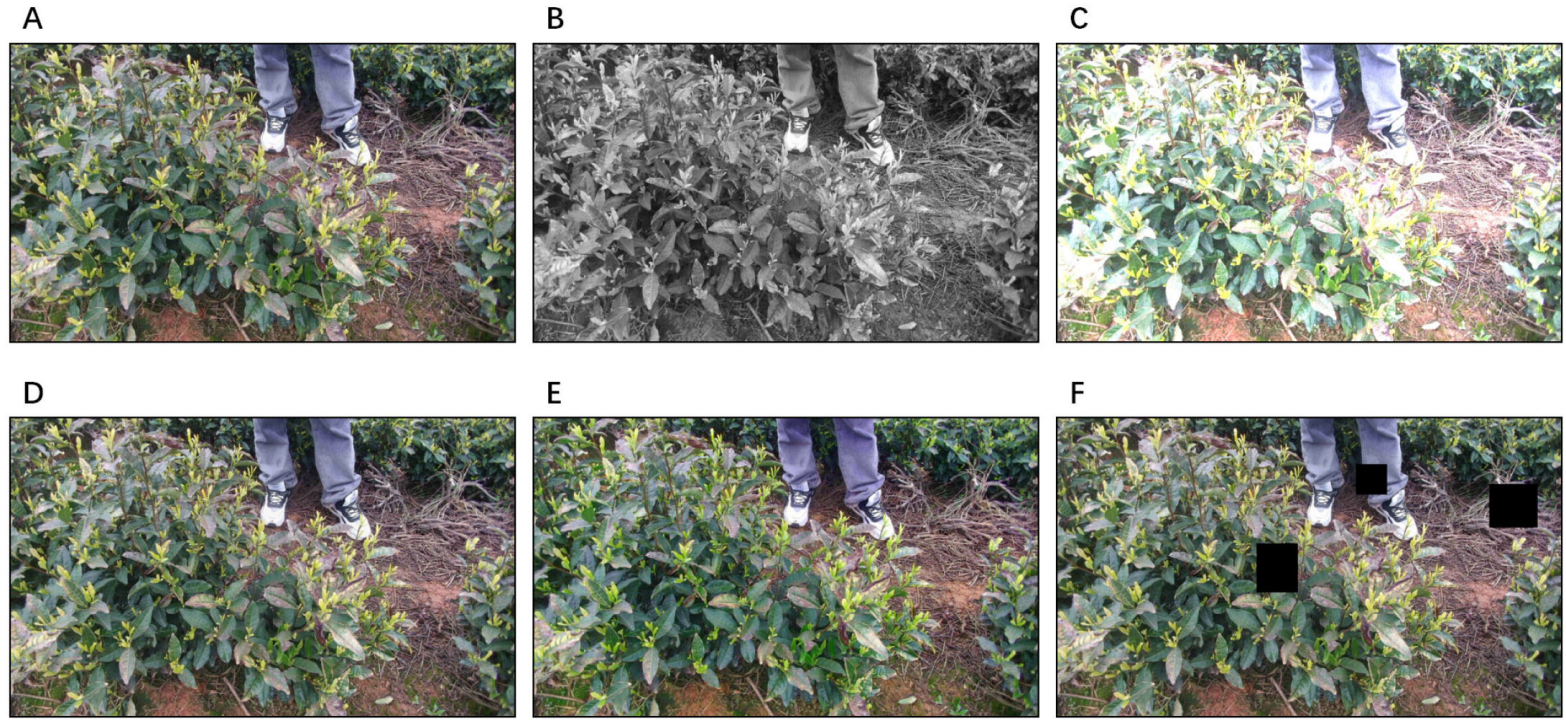
Examples of data augmentation used for tea bud recognition. **(A)** Original image, **(B)** Grayscale conversion, **(C)** Brightness adjustment, **(D)** Gaussian noise addition, **(E)** Hue-saturation adjustment, and **(F)** Cutout.

Meanwhile, to further enhance data diversity and improve the generalization of the YOLO-based tea bud recognition model under the available training data conditions, various data augmentation techniques were applied to the dataset, as shown in **Figure 3**. These augmentations were only used during the training of the two-dimensional recognition model.

Specifically, the applied methods included converting images to grayscale and adjusting brightness to approximate different illumination appearances, rather than explicitly modeling real-world illumination variations. Random noise was also synthetically added to increase data variability and expose the recognition model to diverse perturbations during training. In addition, random hue and saturation adjustments were applied to increase color diversity and improve generalization in complex scenes.

In contrast, for the three-dimensional reconstruction process, data augmentation and noise injection were not applied. Instead, images were further screened based on quality indicators such as lighting conditions and sharpness. Low-quality images—such as those affected by overexposure, underexposure, or low contrast—were manually removed to ensure the geometric stability of camera pose estimation and subsequent neural reconstruction, rather than to optimize any specific reconstruction method. After selection, the number of high-quality images retained in each group ranged from 168 to 225.

### 2.4 2D image processing

#### 2.4.1 YOLOv11

YOLOv11 (Khanam and Hussain, 2024) is the new version of the YOLO series, which combines accuracy, speed, and efficiency in real-time target detection tasks. Compared with its predecessor, YOLOv11 is deeply optimized in terms of network structure and training strategy, significantly enhancing feature extraction capability and inference performance, and is suitable for high-precision detection tasks in complex visual scenes.

The traditional nearest-neighbor interpolation upsampling strategy adopted in YOLOv11 relies on fixed interpolation rules and lacks adaptability to local image features. Since only neighboring pixels are involved in the interpolation process, the effective receptive field is limited, making it difficult to capture broader contextual information. For small tea buds distributed in dense and cluttered plantation scenes, this limitation often leads to blurred spatial details and weakened semantic representation, thereby degrading detection accuracy.

To address this issue, we introduce the lightweight DySample dynamic upsampling module (Liu et al., 2023). DySample employs a point-based adaptive sampling mechanism that dynamically adjusts sampling locations according to local feature responses. Compared with conventional interpolation-based upsampling, DySample significantly reduces parameter count and computational overhead while preserving fine-grained spatial details.

**Figure 4:**
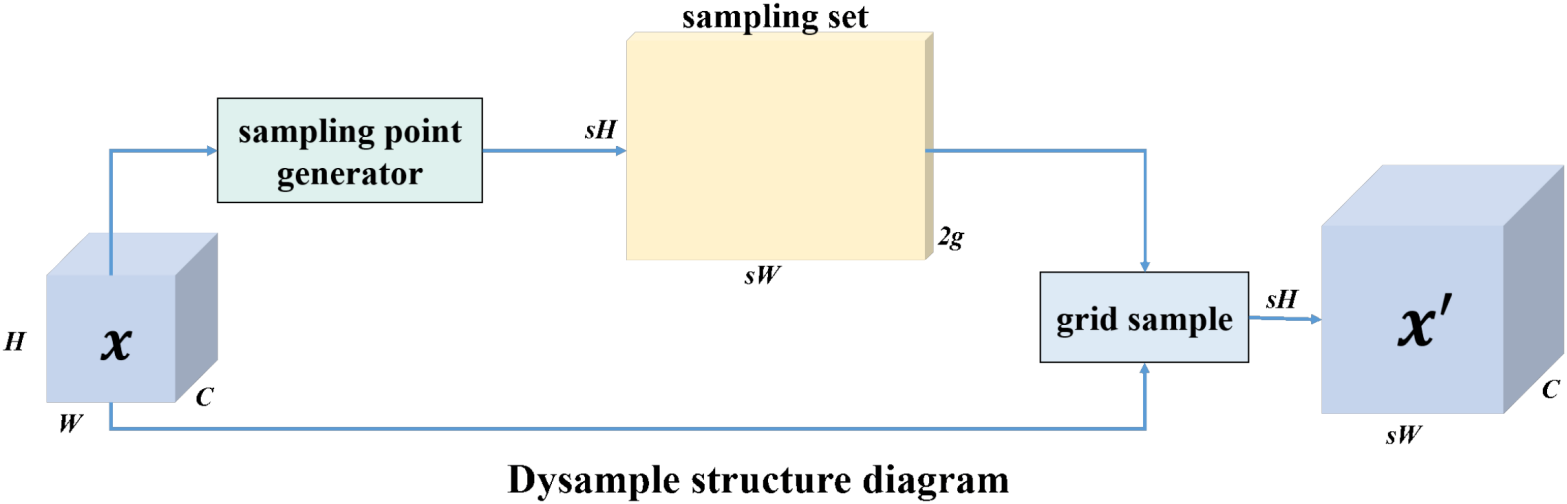
Architecture of the proposed Dysample module. The input feature map is first processed by a sampling point generator to produce a sampling set. The generated sampling grid is then applied through a grid sampling operation to obtain the output feature map with scaled spatial resolution. The height, width, and channel dimensions are preserved in the channel domain while the spatial dimensions are adjusted according to the sampling scale.

By enhancing feature alignment during upsampling, DySample strengthens the model’s focus on tea bud regions and suppresses background interference from leaves and branches. This design improves the robustness of small-object detection in complex natural environments. The specific structure of the DySample module is illustrated in **Figure 4**.

In the backbone of YOLOv11, the C3k2 module is widely adopted in lightweight variants (e.g., n/s) for efficient feature extraction. While shallow C3k2 blocks are effective in capturing local texture and edge information, conventional convolution operations rely on fixed local receptive fields and exhibit strong sensitivity to background clutter. Such locality and noise sensitivity become more pronounced when detecting small or low-contrast targets, such as tea buds under dense occlusion and complex foliage backgrounds, thereby limiting feature discrimination and localization accuracy.

**Figure 5:**
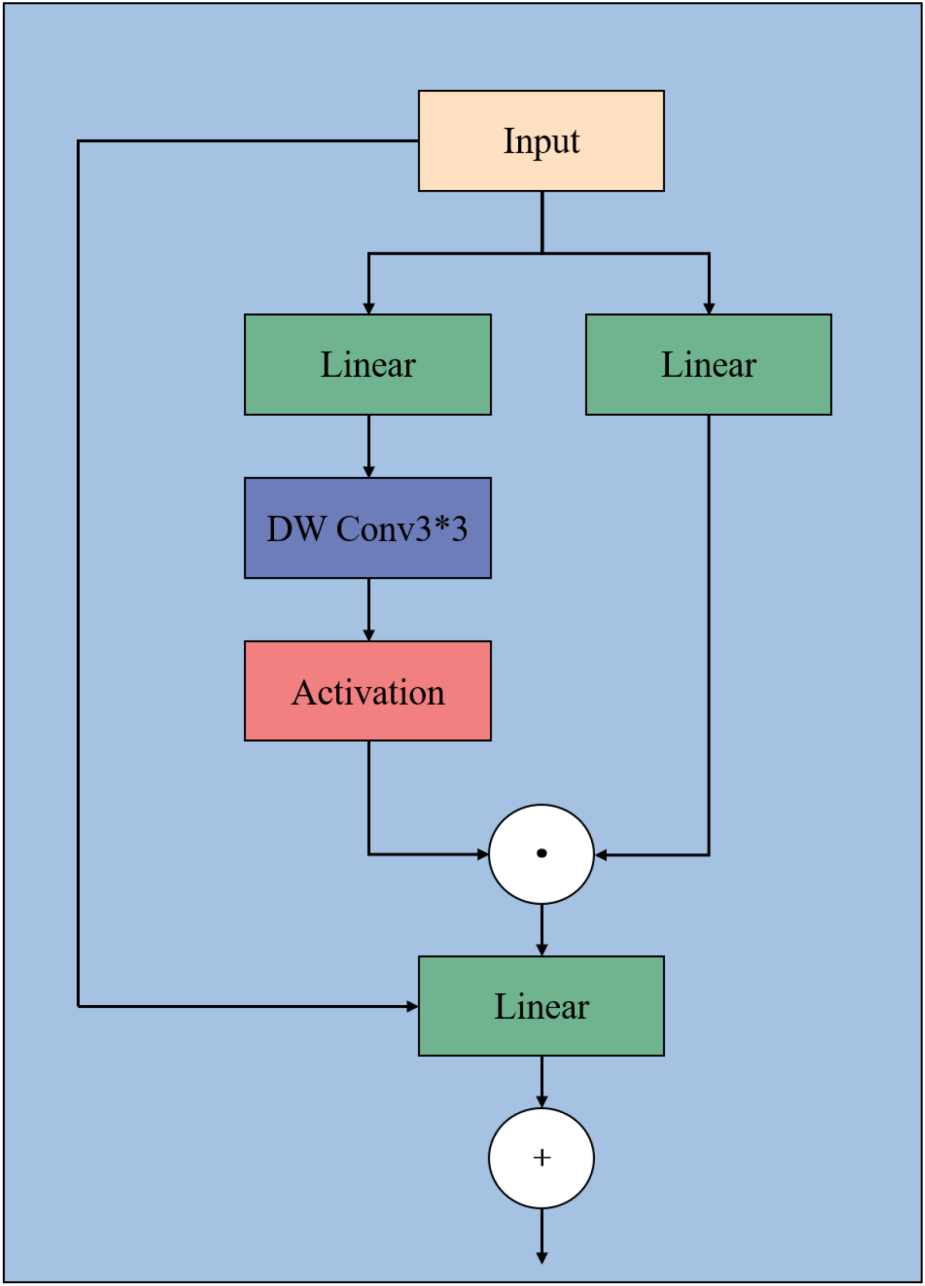
Structure of the Convolutional Gated Linear Unit (CGLU). The module consists of parallel linear projections, a depthwise 3×3 convolution, and a nonlinear activation function, followed by a gated multiplication operation and a residual connection to enhance feature representation.

To alleviate these limitations, we propose a C3k2_DG-SimAM module that introduces lightweight gated feature modulation and parameter-free attention into the original C3k2 design. As illustrated in **Figure 5**, the proposed module consists of two main components: a Bottleneck_DG-SimAM unit and a modified C3k2 aggregation structure.

**Figure 6:**
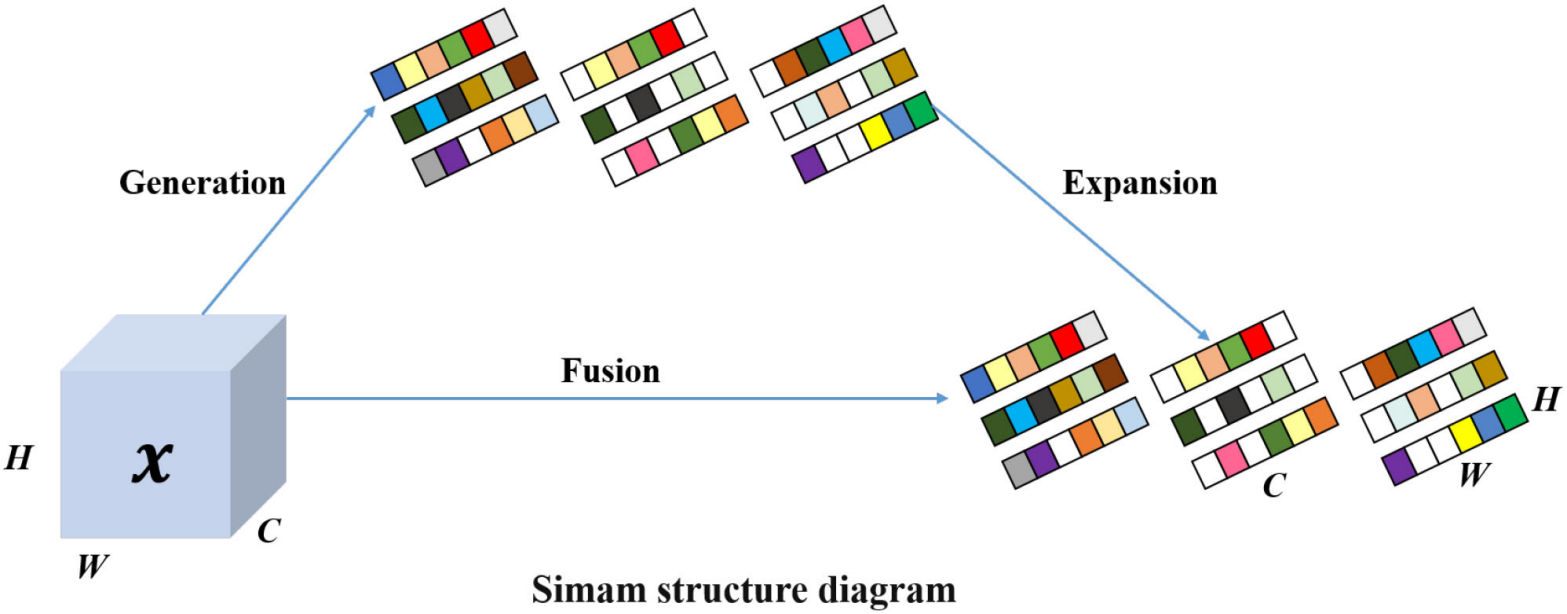
Structure of the Simple Attention Module (SimAM). The module generates attention weights from the input feature map, expands them to match the original feature dimensions, and performs feature fusion to adaptively recalibrate channel-wise responses.

**Figure 7:**
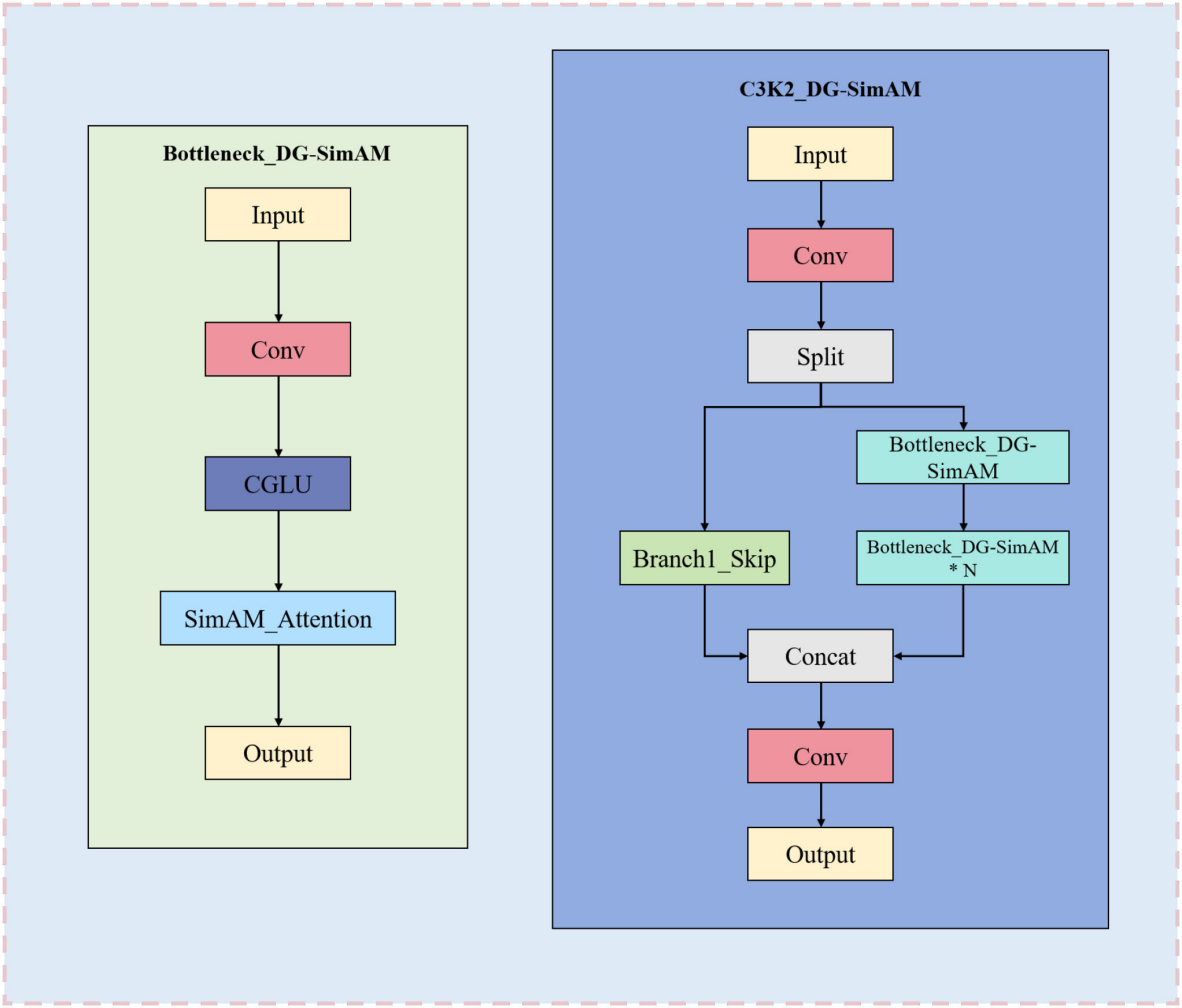
Structure of the proposed C3k2 DG-SimAM module integrating CGLU and SimAM. The left panel illustrates the Bottleneck_DG-SimAM block, which consists of convolution, CGLU, and SimAM attention operations. The right panel shows the overall C3k2-DG-SimAM architecture, including feature splitting, skip connection, stacked Bottleneck_DG-SimAM blocks, concatenation, and final convolution for feature aggregation.

Bottleneck_DG-SimAM. As shown in **Figures 6**, **7**, the Bottleneck_DG-SimAM integrates a Convolutional Gated Linear Unit (CGLU) with the Simple Attention Module (SimAM) (Yang et al., 2021). The CGLU introduces lightweight depthwise gating to suppress redundant activations and selectively emphasize informative feature responses, enabling more robust feature extraction under cluttered background conditions. SimAM further refines feature representations by modeling neuron-level importance across spatial and channel dimensions without introducing additional learnable parameters, thereby enhancing feature discrimination while preserving computational efficiency.

C3k2_DG-SimAM. As illustrated in **Figure 5**, multiple Bottleneck_DG-SimAM units are embedded into the original C3k2 framework following a split–transform–concat strategy. This design preserves the efficient multi-branch feature aggregation characteristics of C3k2 while incorporating gated modulation and attention-enhanced feature refinement. By strengthening the representation of fine-grained and low-contrast features, the proposed C3k2_DG-SimAM module improves robus.

**Figure 8:**
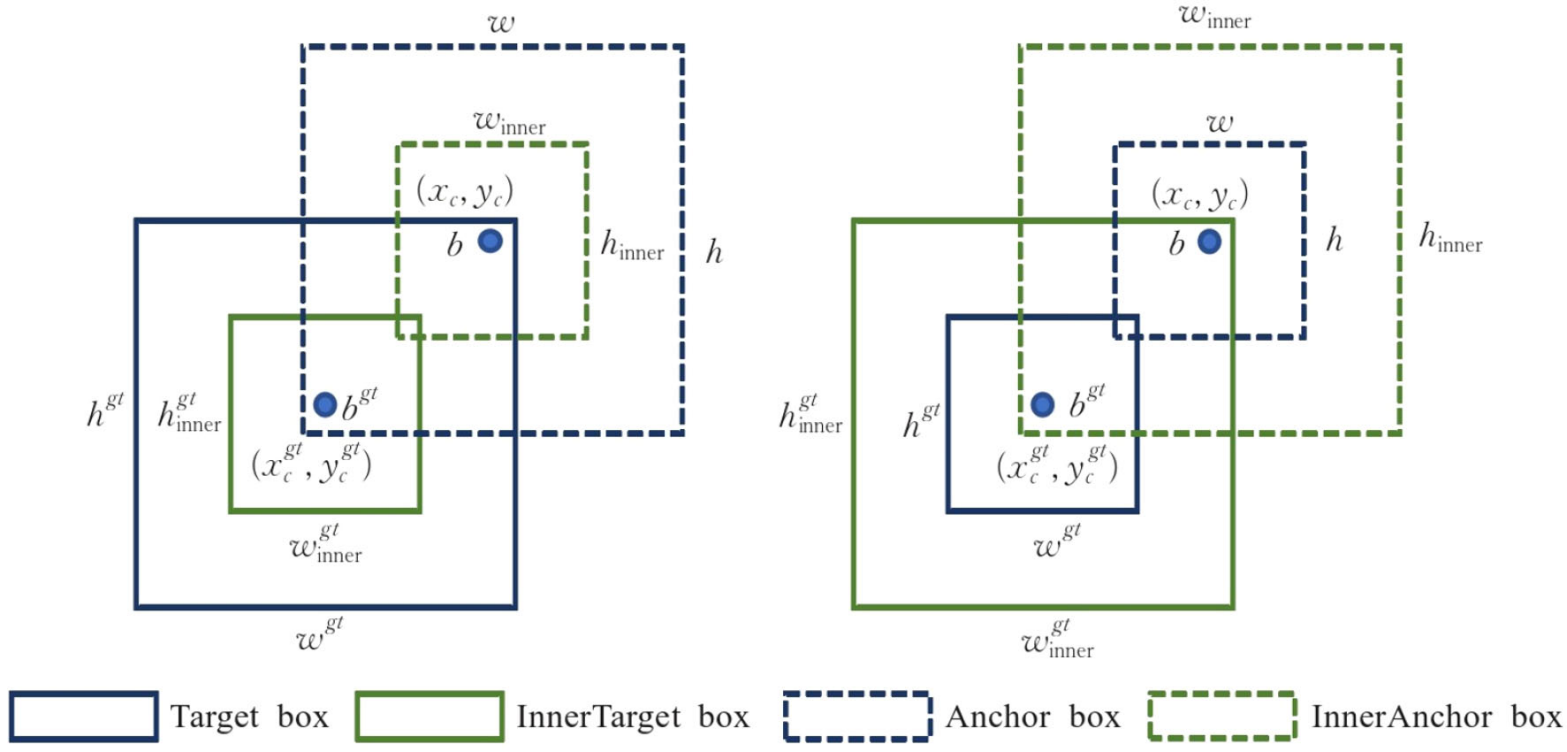
Illustration of the Inner-IoU bounding box relationship. The solid boxes represent the target and inner target regions, while the dashed boxes denote the anchor and inner anchor regions. The diagram shows how the inner bounding boxes are defined within the corresponding target and anchor boxes to guide the Inner-IoU prediction.

In addition, a new loss function, innerIoU (Inner Intersection over Union), is introduced to improve the accuracy of the bounding box for small and difficult-to-locate tea bud targets. Unlike traditional IoU, which only evaluates the overlapping area between the bounding boxes, innerIoU (Zhang et al., 2023b) further considers the degree of alignment between the predicted box and the actual box, thus providing a more rigorous criterion for the localization of the target. The calculation method is shown in **Figure 8**.

Step 1: Compute the inner truth box of the ground by shrinking the GT box with the ratio *r*, as defined in Equation (1):

(1) $$\begin{array}{l} b_{l}^{\text{gt}}=x_{c}^{\text{gt}}-\frac{w^{\text{gt}}\times r}{2},\;b_{r}^{\text{gt}}=x_{c}^{\text{gt}}+\frac{w^{\text{gt}}\times r}{2}\\ b_{t}^{\text{gt}}=y_{c}^{\text{gt}}-\frac{h^{\text{gt}}\times r}{2},\;b_{b}^{\text{gt}}=y_{c}^{\text{gt}}+\frac{h^{\text{gt}}\times r}{2} \end{array}$$

Step 2: Compute the inner anchor box by shrinking the predicted box in the ratio *r*, as defined in Equation (2):

(2) $$\begin{array}{l} b_{l}=x_{c}-\frac{w\times r}{2},\;b_{r}=x_{c}+\frac{w\times r}{2}\\ b_{t}=y_{c}-\frac{h\times r}{2},\;b_{b}=y_{c}+\frac{h\times r}{2} \end{array}$$

Step 3: Calculate the intersection and union areas of the inner boxes, as given in Equations (3) and (4):

(3) $$inter=(\text{min}(b_{r}^{\text{gt}},b_{r})-\text{max}(b_{l}^{\text{gt}},b_{l}))\times (\text{min}(b_{b}^{\text{gt}},b_{b})-\text{max}(b_{t}^{\text{gt}},b_{t}))$$

(4) $$union=(w^{\text{gt}}\times h^{\text{gt}}\times r^{2})+(w\times h\times r^{2})-inter$$

Finally, InnerIoU is given by Equation (5):

(5) $$IoU^{inner}=\frac{inter}{union}$$

**Figure 9:**
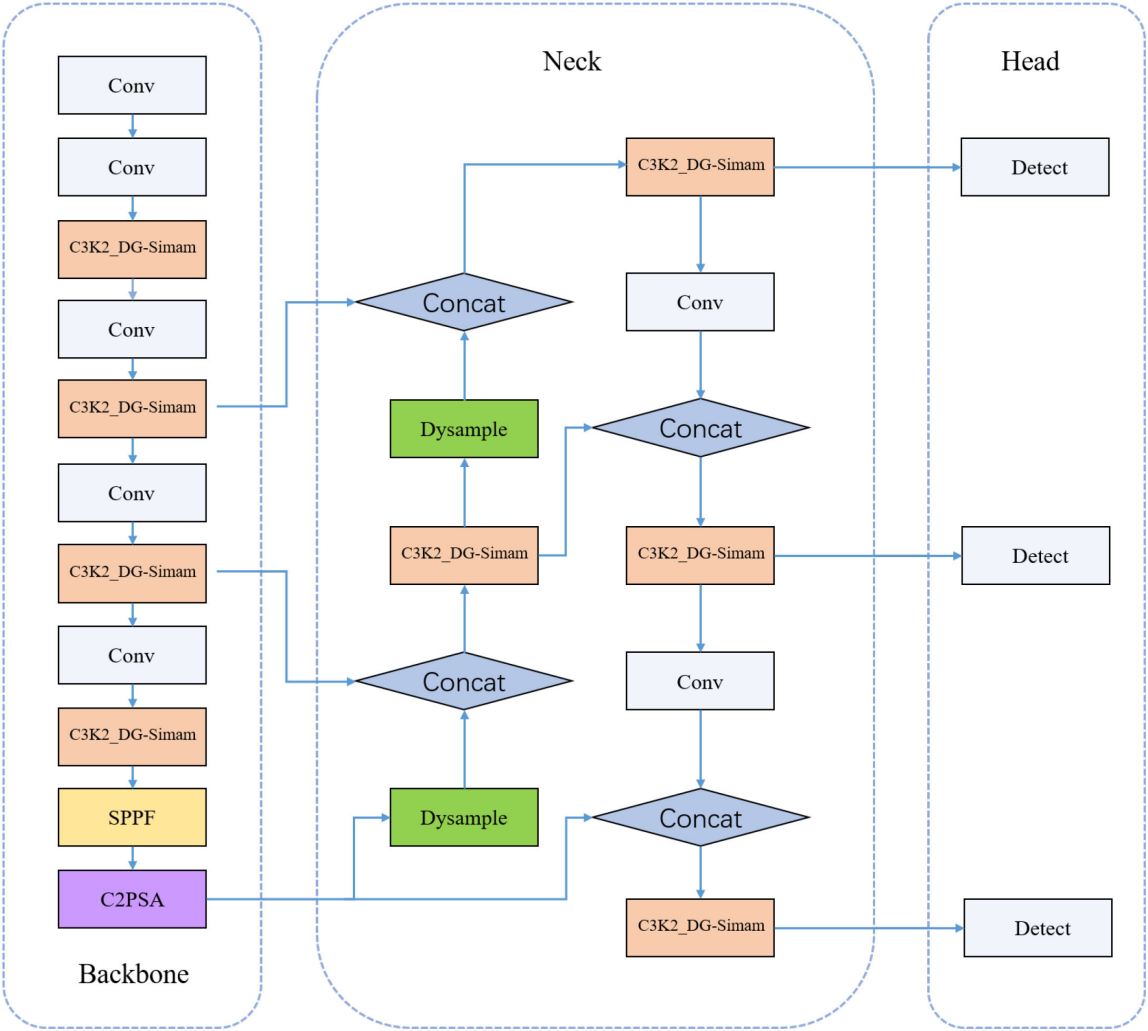
Overall architecture of the proposed tea bud detection model based on YOLOv11. The network incorporates DG-SimAM blocks in the backbone and neck, a Dysample module for feature upsampling, and the Inner-IoU loss for improved localization.

Through the above improvements, the accuracy and robustness of target detection are improved. Its overall structure is shown in **Figure 9**.

#### 2.4.2 Image semantic segmentation

Segment Anything Model 2 (Ravi et al., 2024) (SAM2) is a new generation of image segmentation model introduced by Meta AI, which outperforms its predecessor SAM (Kirillov et al., 2023) in terms of inference efficiency and segmentation performance. SAM2 adopts a more compact and efficient architectural design, which reduces latency and shows better segmentation capability in complex backgrounds and small targets. SAM2 supports flexible segmentation of regions of interest in the form of cues such as dots, bounding boxes, or text.

To realize accurate extraction and background rejection of the tea leaf region, this article combines YOLOv11 and SAM2 to construct an efficient semantic segmentation framework. The method uses the target detection frame provided by YOLOv11 as a hint to guide SAM2 to generate corresponding masks, thus overcoming the limitation that SAM2 only outputs binary masks and lacks semantic annotations, and completing the semantic level segmentation.

To improve the accuracy and robustness of the segmentation mask, a filtering strategy based on the area and segmentation stability is further introduced to eliminate mask regions that do not meet the threshold requirements, to reduce the impact of noise and false detection. At the same time, morphological corrosion operation is used to optimize the edges of the retained mask to remove small artifacts in the area and improve the integrity and clarity of the target contour. Finally, the optimized mask is converted to a JPG format image to complete the semantic segmentation process.

This method not only significantly reduces the cost of manual annotation, but also improves the segmentation accuracy of tea leaf images in complex environments, which provides a reliable basis for subsequent target analysis and 3D reconstruction.

#### 2.4.3 Depth estimation

The task of three-dimensional reconstruction of tea leaves poses significant challenges to traditional image-based matching methods due to their intricate structure, severe leaf occlusion, high surface texture repetition, and susceptibility to lighting conditions. To address this, the incorporation of high-quality depth maps can significantly enhance the geometric accuracy and robustness of the reconstruction. Depth maps provide spatial location information for each pixel, effectively alleviating matching difficulties caused by occlusion, texture loss, or sparse features, particularly for natural objects like tea stems and leaves that exhibit non-rigid shapes and locally repetitive structures.

To achieve the depth estimation of the images, the Depth Anything V2 model (Yang et al., 2024a) is used in this paper. This model is a generalized depth estimation framework based on the vision-based model, which is capable of high-quality depth prediction for a wide range of natural images in unsupervised and weakly supervised scenarios. Depth Anything V2 architecturally combines an image encoder and a multiscale depth decoder, with good cross-scene generalization ability and robustness to complex lighting and texture occlusion. By applying this model to multi-view images, it can provide dense and continuous depth information for subsequent 3D reconstruction tasks, which provides strong support for improving the training accuracy and geometric restoration capability of NeRF.

### 2.5 Tea tree 3D reconstruction

To reconstruct high-quality three-dimensional representations of tea trees, we adopt the Nerfacto framework within NeRFStudio (Mildenhall et al., 2021; Zhang et al., 2021). Nerfacto provides an efficient implementation of neural radiance fields by optimizing the sampling strategy and network design, achieving a favorable balance between rendering quality and computational efficiency. This makes it suitable for high-resolution reconstruction of complex plant structures under field conditions.

To further enhance geometric fidelity and accelerate convergence, monocular depth priors are incorporated during training through a depth-supervised loss, enabling the model to better capture fine-scale structures such as thin branches and densely clustered tea buds. In addition, a semantic branch is introduced to extend Nerfacto from pure appearance modeling to semantic-aware 3D reconstruction. The semantic field predicts tea bud probabilities as a function of spatial location, allowing semantic information to be consistently aligned with reconstructed geometry. Standard volumetric rendering formulations for color, depth, and semantics are consistent with the original NeRF framework and are therefore not detailed here.

### 2.6 Tea bud point cloud processing

Unlike most existing NeRF-based agricultural studies that primarily focus on visual reconstruction or phenotypic analysis, this section introduces a harvesting-oriented three-dimensional perception strategy. By explicitly processing bud-level semantic point clouds, the proposed method bridges three-dimensional reconstruction with practical harvesting tasks, including tea bud counting and harvesting-oriented candidate point estimation. The harvesting-oriented candidate point is obtained from bud-level semantic point clouds and provides a three-dimensional spatial reference for subsequent harvesting planning. This design enables the extraction of actionable spatial cues from reconstructed point clouds, establishing an effective perception foundation for harvesting-oriented applications.

#### 2.6.1 Point cloud processing and bud-level clustering

Before clustering analysis, the reconstructed three-dimensional point cloud is first preprocessed to remove isolated noise points. Specifically, a radius-based filtering strategy is applied, in which points with an insufficient number of neighboring points within a predefined radius are discarded. This step effectively reduces spurious points introduced during reconstruction and improves the reliability of subsequent clustering and geometric analysis.

To enable reliable tea bud instance separation and counting, a density-based spatial clustering algorithm (DBSCAN) (Schubert et al., 2017) is adopted. DBSCAN identifies core points by evaluating local point density within a predefined neighborhood and groups density-reachable points into clusters, while automatically rejecting sparse outliers. Through this process, individual tea buds are separated into distinct three-dimensional clusters, each corresponding to a candidate tea bud instance.

For clusters with a small spatial scale, an additional merging strategy is applied. If the Euclidean distance between the centroids of two clusters is smaller than the average radius of a tea bud, the clusters are considered to belong to the same tea bud structure and are merged accordingly. The remaining tiny clusters are further examined by estimating their volumetric size. Clusters whose volumes are significantly smaller than those of typical tea bud clusters are regarded as non-target structures and are removed. After these steps, the resulting bud-level clusters provide a robust basis for tea bud counting and subsequent spatial analysis.

All hyperparameters that directly affect the reproducibility of the proposed three-dimensional perception pipeline, including density-based clustering, semantic filtering thresholds, loss weighting, and geometric estimation criteria, are summarized in the **Supplementary Material** (**Supplementary Table 1**).

#### 2.6.2 Harvesting-oriented candidate point estimation

Based on the bud-level point cloud clusters obtained in the previous step, a harvesting-oriented candidate point is estimated for each tea bud. This estimation aims to provide a stable three-dimensional spatial reference derived from perception, rather than a final robot-executable cutting command.

For each tea bud cluster, all points are first sorted along the Z-axis (vertical direction), and a subset of points within the lowest height range (e.g., the lowest 5%) is extracted. Rather than assuming a fixed anatomical stem–bud junction, these points are used as a geometric approximation of the basal-side surface region under a harvesting-oriented prior. Such a proxy often corresponds to the attachment side of the bud cluster when the basal structure is visible in the reconstructed point cloud, but it does not rely on the assumption that all buds grow strictly upward in the global coordinate system. Consequently, laterally growing or obliquely oriented buds can still be handled, as the candidate point is not defined as an anatomically exact junction.

To mitigate the influence of noise and isolated extreme points, a robust estimation strategy is employed. Specifically, RANSAC-based plane fitting (Derpanis, 2010) or local geometric center estimation is applied to the selected lower subset, providing a local geometric prior for basal-side localization. The geometric center of the fitted plane (or the estimated local center when plane fitting is degenerate) is taken as the candidate point. Compared with directly selecting individual extreme points, this strategy yields improved stability and repeatability under point cloud perturbations.

**Figure 10:**
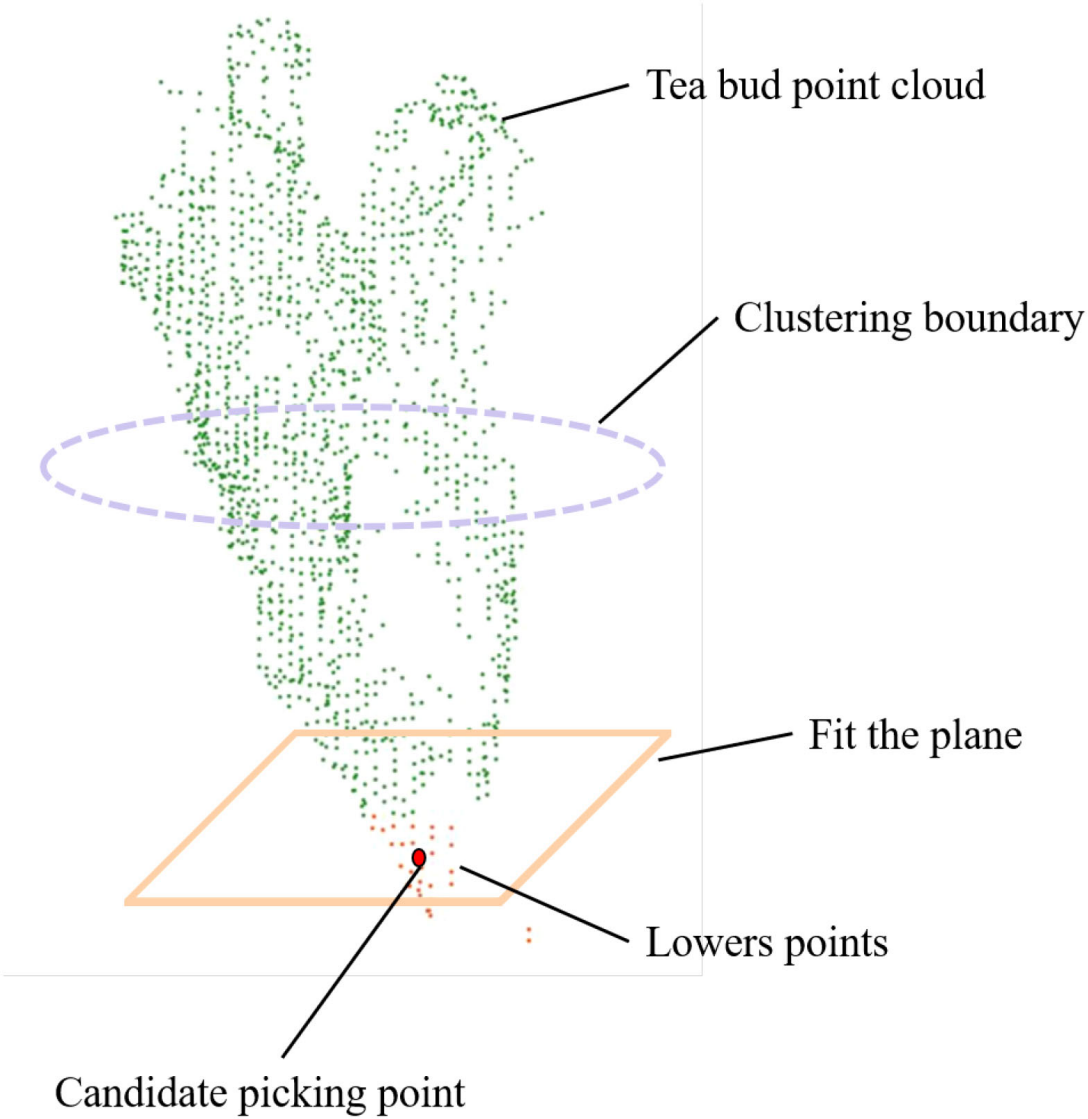
Harvesting-oriented candidate picking point estimation from the tea bud point cloud.

The estimated candidate point is derived purely from three-dimensional perception and represents a harvesting-oriented guidance cue rather than a ground-truth stem–bud junction. It provides a consistent spatial reference for tea bud localization and can support downstream harvesting planning. Specific implementations related to cutting execution, such as end-effector configuration and motion control, are system-dependent and therefore beyond the scope of this study. An illustrative example of the candidate point estimation process is shown in **Figure 10**.

## 3 Results

### 3.1 Detection modeling of tea buds

The detection models in this study were evaluated using precision (P), recall (R), and mean accuracy (mAP), where P denotes the proportion of accurate predictions in all prediction examples and R denotes the proportion of accurate predictions in all true examples. mAP denotes the composite accuracy metric used to evaluate the detection models. The formulas for the computation of P, R, and mAP are shown in Equations (6–8).

(6) $$P=\frac{TP}{TP+FP}$$

(7) $$R=\frac{TP}{TP+FN}$$

(8) $$\text{mAP}=\frac{\sum_{i=1}^{N}P(R_{i})\cdot \text{Δ}R_{i}}{N}$$

TP: number of positive samples predicted as positive samples.

FP: Number of negative samples predicted as positive samples.

FN: number of positive samples predicted as negative samples.

N: denotes the number of bud types detected (only one type of tea bud is studied in this paper, so N is equal to 1).

It can be seen from **Table 1** that each component, including DySample, DG-SimAM, and InnerIoU, contributes positively to the overall detection performance. Specifically, DySample mainly improves detection coverage, as reflected by the increase in mAP@50, while InnerIoU enhances localization accuracy, resulting in a higher mAP@50:95. DG-SimAM further improves recall and robustness under complex backgrounds.

**Table 1:** Results of ablation experiments.

Number	+DySample	+DG-SimAM	+InnerIoU	P	R	mAP@50	mAP@50:95	Params	GFLOPs
0				0.776	0.801	0.869	0.624	2 938 835	6.7
1	✓			0.813	0.839	0.907	0.567	2 602 387	6.5
2		✓		0.789	0.826	0.889	0.603	3 157 075	7.1
3			✓	0.800	0.829	0.897	0.631	2 938 835	6.7
4	✓		✓	0.819	0.841	0.911	0.582	2 602 387	6.5
5		✓	✓	0.799	0.837	0.901	0.621	3 157 075	7.1
6	✓	✓		0.815	0.837	0.909	0.605	3 169 427	7.1
7	✓	✓	✓	0.827	0.843	0.917	0.651	3 169 427	7.1

When combined, the proposed model achieves the best results, with Precision, Recall, mAP@50, and mAP@50:95 reaching 0.827, 0.843, 0.917, and 0.651, respectively. Compared to the baseline YOLOv11n, these values represent improvements of 5.1% in Precision, 4.2% in Recall, 4.8% in mAP@50, and 2.7% in mAP@50:95, while maintaining comparable parameters and computational cost (GFLOPs). This indicates that the introduced modules not only enhance feature extraction and localization accuracy, but also achieve a favorable trade-off between accuracy and efficiency.

**Figure 11:**
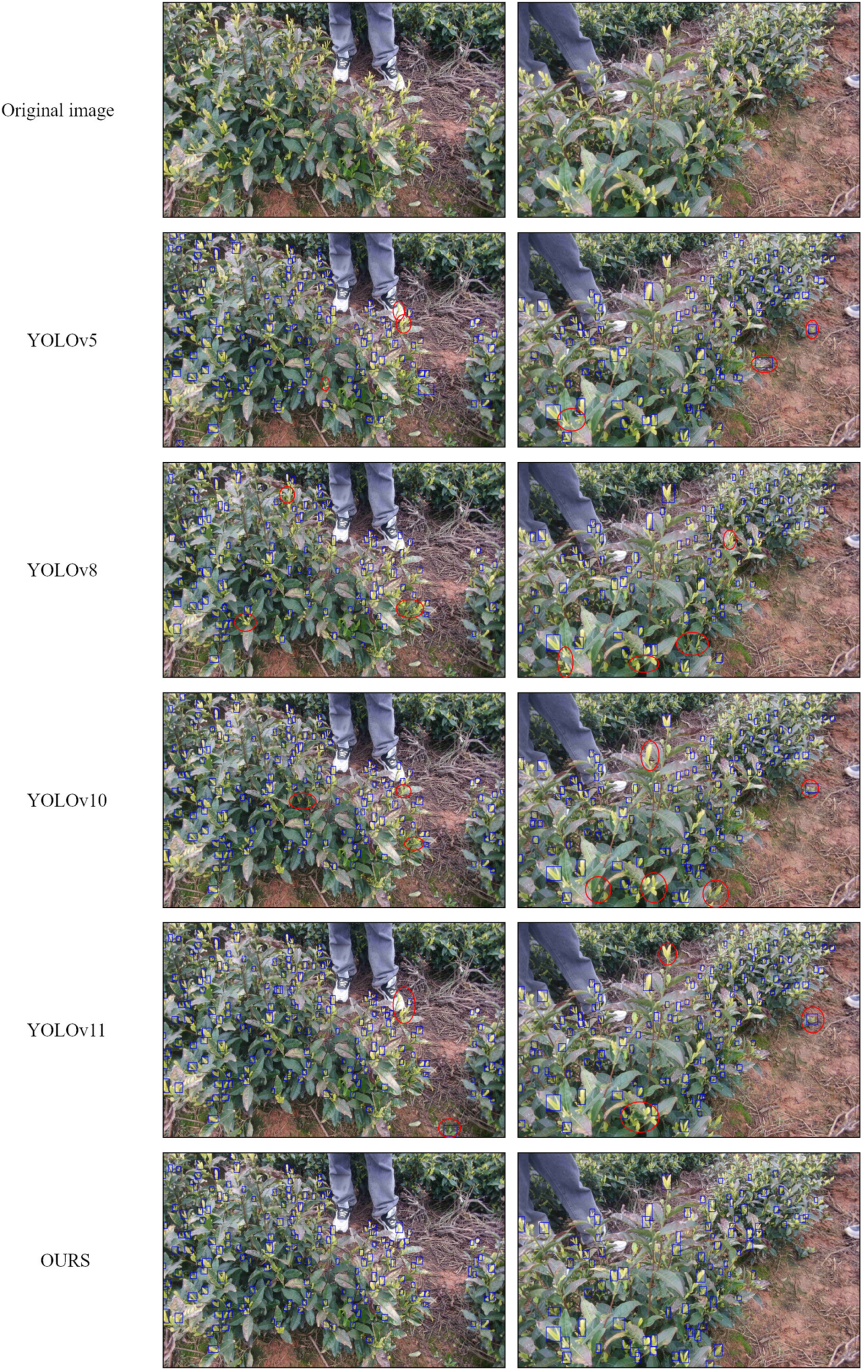
Tea bud detection results. Red ovals mark misidentified and missed tea buds.

Furthermore, as summarized in **Table 2** and illustrated in **Figure 11**, the proposed model consistently outperforms other YOLO variants across all evaluation metrics. Although YOLOv5n, YOLOv8n, and YOLOv10n demonstrate competitive performance, the improved YOLOv11-based model achieves superior detection accuracy and localization stability. In particular, the improvement in mAP@50:95 indicates more precise bounding box regression under stricter IoU thresholds, which is crucial for detecting small, densely distributed, and partially occluded tea buds in complex tea garden environments.

**Table 2:** Comparison of the improved model with other models.

Model	P	R	mAP@50	mAP@50:95	Params	GFLOPs
YOLOv5n	0.759	0.786	0.851	0.554	2,188,019	5.9
YOLOv8n	0.778	0.790	0.862	0.550	2,690,403	6.9
YOLOv10n	0.789	0.805	0.875	0.599	2,707,430	8.4
YOLOv11n	0.776	0.801	0.869	0.624	2,938,835	6.7
Ours	0.827	0.843	0.917	0.651	3,169,427	7.1

Accurate localization is especially important in the proposed pipeline, as the detection results are directly used as prompts to guide SAM2 for fine-grained semantic segmentation. Inaccurate or shifted bounding boxes may propagate errors to the segmentation stage, leading to incomplete or imprecise tea bud masks.

### 3.2 2D image segmentation

To achieve semantic segmentation of tea buds, we employed three methods: YOLO+SAM, YOLO+SAM2, and a self-trained U-Net (Ronneberger et al., 2015). In addition, a representative single-stage instance segmentation model, Mask R-CNN, was included as a baseline for comparison. The U-Net training data set consisted of two parts: 60 manually annotated images from our captured data set and 30 additional images refined from the YOLO+SAM2 segmentation results. Data augmentation techniques, including color transformation (brightness, contrast, and saturation adjustments within 0.5–1.5), image flipping, rotation, scaling, and noise injection, were applied to improve robustness. The U-Net model was implemented in PyTorch.

The comparative performance of all methods is summarized in **Table 3**. YOLO+SAM2 achieved the highest IoU (0.640) and Dice coefficient (0.779) while maintaining a reasonable inference time (0.511 s). YOLO+SAM closely followed with an IoU of 0.629 and a Dice score of 0.771. U-Net was significantly faster (0.013 s) but less accurate (IoU 0.597, Dice 0.747). Mask R-CNN exhibited lower segmentation accuracy (IoU 0.578, Dice 0.725) while remaining computationally efficient (0.028 s).

**Table 3:** Comparison of different segmentation methods for tea bud images.

Model	IoU	Dice	Inference time (s)
YOLO + SAM	0.629	0.771	0.523
YOLO + SAM2	0.640	0.779	0.511
U-Net	0.597	0.747	0.013
Mask R-CNN	0.578	0.725	0.028

**Figure 12:**
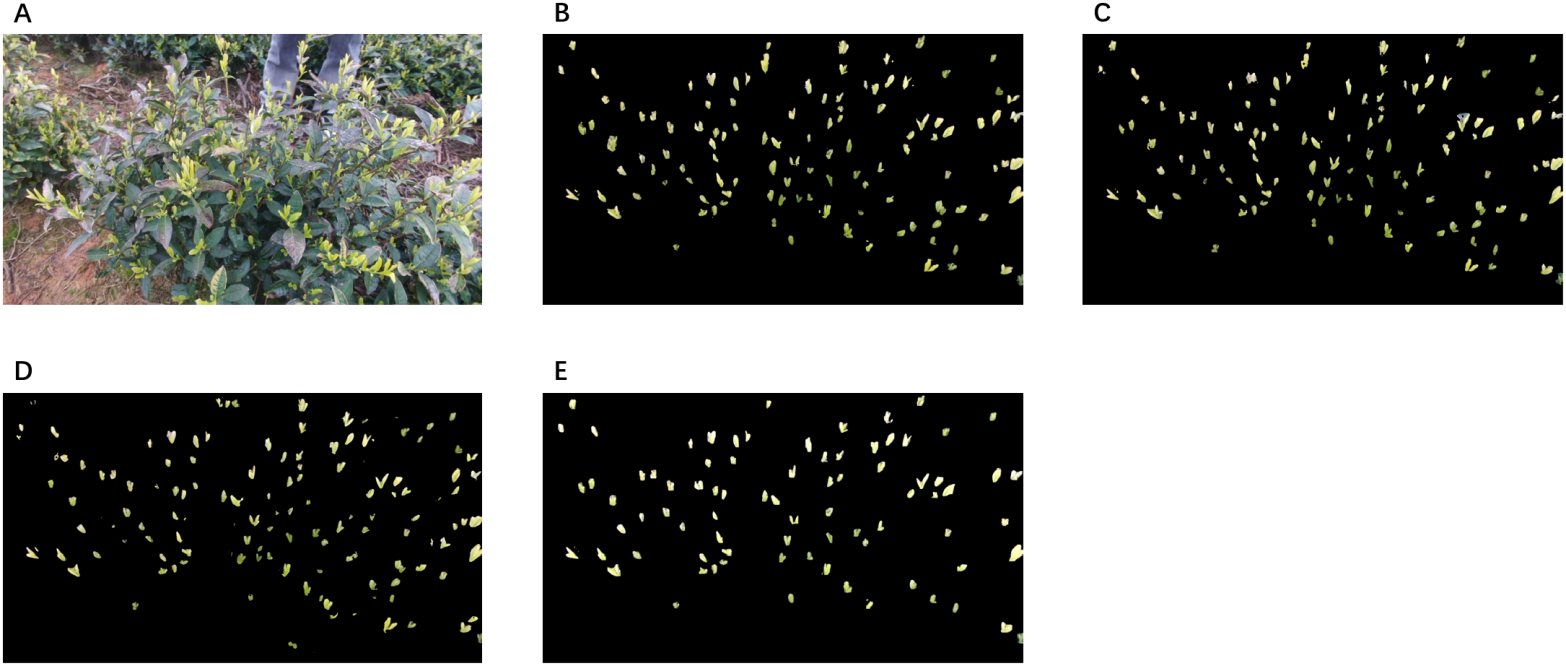
Examples of segmentation results for different models. **(A)** Original **(B)** SAM2 **(C)** SAM **(D)** U-Net **(E)** Mask R-CNN.

As shown in **Figure 12**, YOLO+SAM2 produces more precise segmentation of small and occluded tea buds, with improved boundary delineation compared to SAM, benefiting from its more efficient attention mechanism and lighter architecture. YOLO+SAM also shows competitive performance, though with slightly reduced accuracy. U-Net, despite its high speed and independence from cue information, tends to over-segment tea leaves and struggles with fine or deeply embedded tea buds under complex canopy conditions. Mask R-CNN, while capable of directly predicting instance-level masks, often suffers from incomplete or fragmented bud segmentation in densely occluded regions, which limits its effectiveness in this scenario.

Overall, the quantitative results in **Table 3** and the qualitative comparisons in **Figure 12** demonstrate that YOLO+SAM2 achieves the best balance between segmentation accuracy and inference efficiency. Although segmentation errors remain under realistic harvesting conditions—mainly due to boundary ambiguity and severe occlusion—the improved boundary precision of YOLO+SAM2 leads to more compact and consistent semantic point clusters, which is beneficial for downstream tasks such as tea bud counting and candidate picking-point estimation in harvesting-oriented 3D perception.

### 3.3 Tea tree 3D reconstruction

Semantic 3D reconstruction is a key part of the process. The classical 3D representations of point clouds, meshes, and voxels are limited in their effectiveness in representing complex scenes and are limited in representing fine-grained geometry and view-dependent appearance in densely cluttered canopies, motivating the use of NeRF to model radiance and density as continuous fields. With body rendering techniques, NeRF can generate high-quality images from arbitrary viewpoints. Nerfstudio is an open-source framework that provides many frameworks for NeRF. We extend the Nerfacto method by adding a semantic layer that maps points and viewpoint directions in 3D space to semantic information as well, which can be viewed as a semantic field.

**Figure 13:**
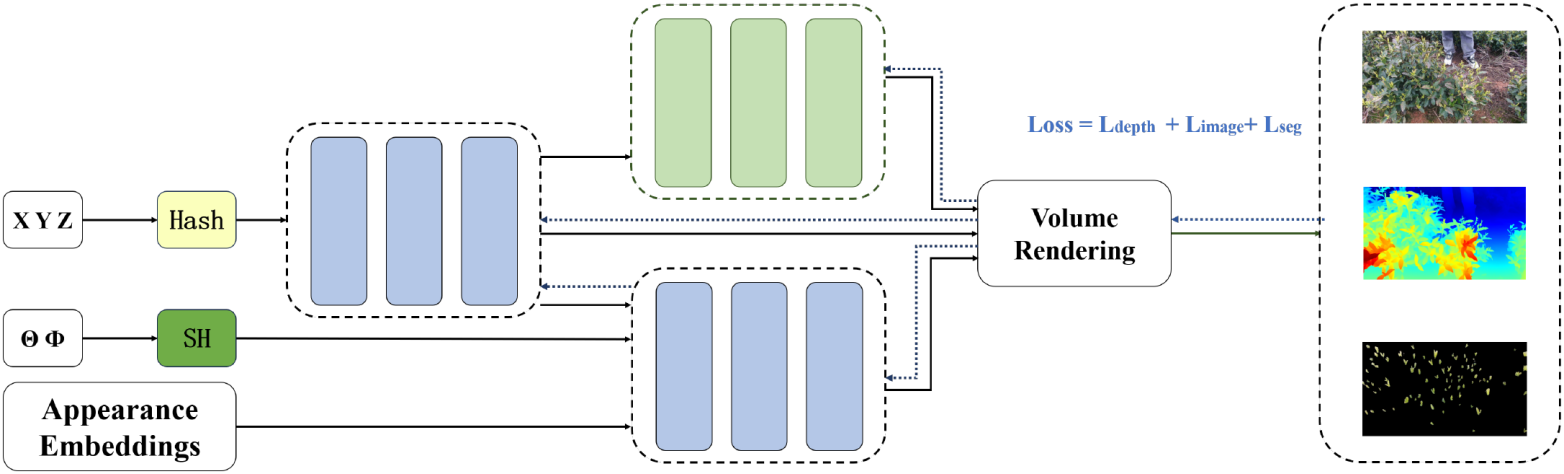
Network architecture of the proposed TeaNeRF reconstruction framework. The model takes spatial coordinates and appearance embeddings as input, applies hash encoding and multi-layer perceptrons, and performs volume rendering to generate RGB images, depth maps, and segmentation outputs. The training loss consists of depth, image reconstruction, and segmentation supervision terms.

As shown in **Figure 13**, the proposed 3D reconstruction framework for tea trees adopts multimodal encoding and collaborative neural network modeling to establish an efficient mapping between 3D space and 2D images at the reconstruction level. Specifically, spatial points (*x, y, z*) along each ray and their corresponding view direction vectors d are encoded using hash encoding and spherical harmonic (SH) encoding for feature extraction, respectively. Hash encoding effectively reduces the computational overhead of traditional positional encoding and supports high-resolution geometric modeling, while SH encoding captures view-dependent orientation information to improve the modeling accuracy of lighting and material appearance. The fused appearance embedding vectors are nonlinearly mapped by a multilayer perceptron (MLP) to predict volume density (*σ*) and color (RGB), and novel views are rendered via volume rendering. This decoupled architecture improves reconstruction efficiency compared to conventional NeRF formulations while maintaining reconstruction quality. On an NVIDIA RTX 4090 (24GB VRAM), training on a set of 3840×2160 resolution images takes approximately 5 minutes per tea tree. This runtime reflects an offline, per-tree reconstruction setting focused on high-fidelity geometric and semantic perception, rather than real-time harvesting execution.

For completeness, an approximate runtime breakdown of the proposed pipeline is provided in the **Supplementary Material** (**Supplementary Table 2**).

In the training phase, the photometric loss is calculated as defined in Equation (9) by comparing the difference between the predicted color of each ray and the real pixel RGB value, thus optimizing the color reconstruction performance of the model. For semantic rendering, the semantic loss is defined in Equation (10), where the model estimates the probability of each pixel belonging to the tea bud or the background based on the prediction density and introduces the binary cross-entropy loss to enhance semantic segmentation accuracy. Meanwhile, to enhance the model’s ability to model the geometric structure of the scene, the depth-supervised loss is defined in Equation (11), improving the accuracy and robustness of 3D reconstruction by constraining the consistency between the predicted depth and the true depth.

(9) $$L_{\text{image}}=\frac{1}{\left|R\right|}||C(r)-\hat{C}(r){||}_{2}^{2}$$

(10) $$L_{\text{sem}}=\frac{1}{\left|R\right|}\sum_{r\in R}[p(r)\log\hat{p}(r)+(1-p(r))\;\text{log}(1-\hat{p}(r))]$$

(11) $$L_{\text{depth}}=\frac{1}{\left|R\right|}\sum_{r\in R}||D(r)-\hat{D}{\left(r)|\right|}_{1}$$

The overall training objective is defined in Equation (12):

(12) $$L=L_{\text{image}}+\lambda_{\text{sem}}\cdot L_{\text{sem}}+\lambda_{\text{depth}}\cdot L_{\text{depth}}$$

**Figure 14:**
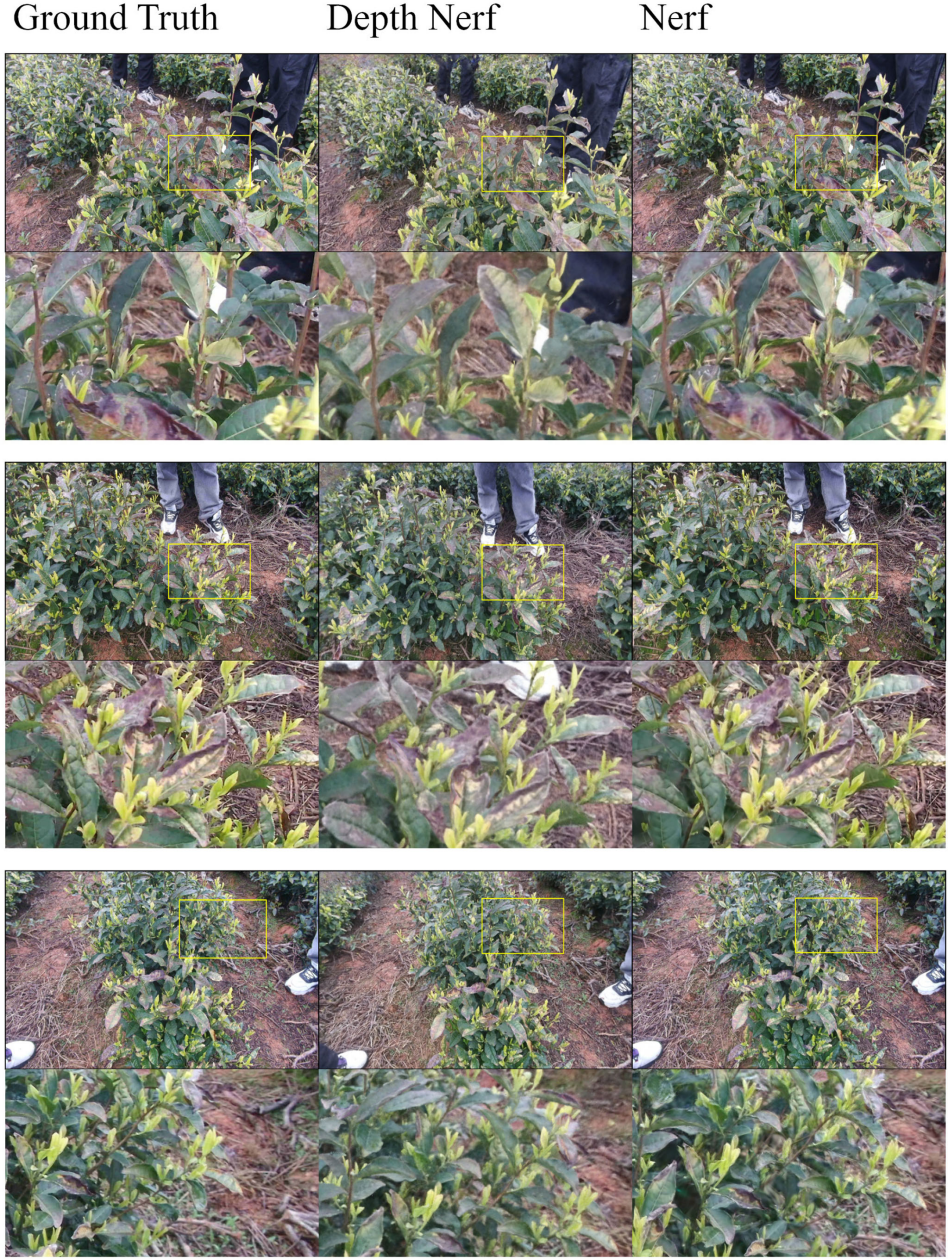
Qualitative NeRF rendering results. The top rows show rendered views of tea plants, with highlighted regions indicating areas of interest. The bottom rows present zoomed-in views for detailed comparison of reconstructed structures.

In this study, NeRF is applied to achieve 3D reconstruction of tea trees based on multi-view images. The model synthesizes geometrically consistent views from different perspectives, thereby generating dense point clouds that support subsequent structural and semantic analysis. Nevertheless, conventional NeRF often produces rendered images that appear less sharp than the original photos, particularly in regions with densely distributed tea buds. This is mainly because NeRF prioritizes geometric consistency and multi-view synthesis fidelity, while fine-grained details may accumulate rendering errors and lead to local blurring. To overcome this limitation, depth information was incorporated into NeRF (Depth-NeRF), allowing more accurate depiction of subtle tea bud structures, clearer leaf-edge contours, and improved overall sharpness, as illustrated in **Figure 14**.

To systematically evaluate the contribution of monocular depth priors to Neural Radiance Fields (NeRF) for reconstructing plant-like natural objects, three tea trees with distinct morphologies (denoted as Tree1, Tree2, and Tree3) were selected. The baseline NeRFacto model was compared with a depth-supervised variant, Depth-NeRF, which incorporates monocular depth priors estimated using Depth Anything v2 during training iterations ranging from 2,000 to 30,000. Reconstruction performance was evaluated using Peak Signal-to-Noise Ratio (PSNR), Structural Similarity Index (SSIM), and Learned Perceptual Image Patch Similarity (LPIPS), providing a comprehensive assessment of both image fidelity and perceptual quality.

As shown in **Table 4**, Depth-NeRF consistently outperforms NeRFacto in all three test cases. In the Tree1 scene, Depth-NeRF achieves a PSNR of 24.08, SSIM of 0.704, and LPIPS of 0.209 at 30,000 iterations, compared to 23.91, 0.698, and 0.247 for NeRFacto, respectively. Although the absolute PSNR and SSIM improvements appear modest, the substantial reduction in LPIPS highlights a clear enhancement in perceptual realism and fine structural detail. A similar trend is observed for Tree2 and Tree3, with Tree3 showing the most significant gain (LPIPS reduced from 0.599 to 0.405), indicating improved robustness in handling occlusion, texture repetition, and complex leaf structures. However, residual reconstruction uncertainty remains in densely occluded regions and around thin structures, where monocular depth ambiguity may cause local geometric blur or incomplete surfaces that can propagate to subsequent semantic point cloud extraction.

**Table 4:** Evaluation results for different iteration counts.

Tree	Iterations	NeRF			Depth-NeRF		
		PSNR	SSIM	LPIPS	PSNR	SSIM	LPIPS
Tree1	2000	21.78	0.543	0.493	22.11	0.569	0.473
	4000	22.37	0.589	0.411	22.67	0.605	0.393
	8000	23.10	0.631	0.334	23.43	0.652	0.323
	10000	23.43	0.655	0.288	23.62	0.674	0.303
	30000	23.91	0.698	0.247	24.08	0.704	0.209
Tree2	2000	17.07	0.399	0.771	17.21	0.405	0.747
	4000	17.17	0.404	0.749	17.43	0.413	0.710
	8000	17.43	0.410	0.722	17.68	0.421	0.678
	10000	17.79	0.425	0.677	17.81	0.428	0.657
	30000	17.88	0.431	0.681	18.13	0.447	0.572
Tree3	2000	18.70	0.456	0.749	19.44	0.485	0.635
	4000	19.45	0.486	0.649	19.60	0.493	0.620
	8000	19.46	0.485	0.633	20.15	0.551	0.580
	10000	20.05	0.501	0.599	20.83	0.613	0.405
	30000	20.90	0.599	0.399	21.03	0.622	0.394

Another important observation is that Depth-NeRF exhibits faster convergence during the early training phase (2k–8k iterations), producing sharper and less noisy reconstructions compared to the baseline. This demonstrates that monocular depth priors not only improve final reconstruction quality but also stabilize training and accelerate geometry learning under limited iterations.

**Figure 15:**
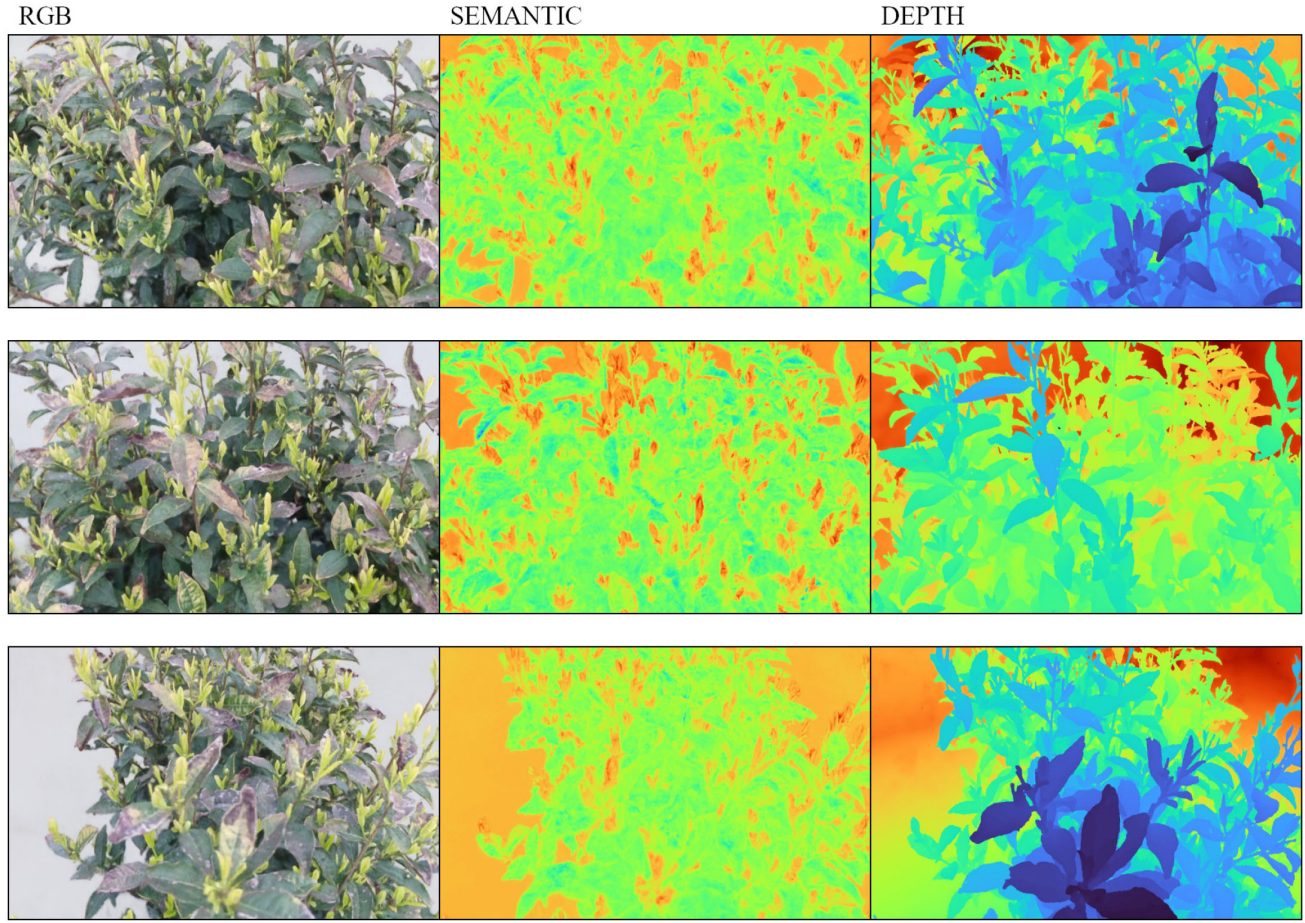
Volume rendering results of the proposed TeaNeRF framework. From left to right, the columns show the rendered RGB images, semantic predictions, and depth maps under different viewpoints.

**Figure 15** further illustrates the qualitative improvements. The RGB renderings generated by Depth-NeRF under natural lighting accurately restore the tea tree morphology, with clearer branch-leaf hierarchies than NeRFacto. In semantic rendering, tea buds (highlighted in red) are accurately separated from surrounding leaves (blue-green), verifying the capability of Depth-NeRF to achieve fine-grained semantic segmentation in cluttered environments. The depth maps reveal an enhanced contrast between the body of the tea tree (blue-to-yellow gradient) and the background (orange-red), strengthening the model’s ability to capture meaningful depth differences.

Overall, the integration of monocular depth priors significantly improves both perceptual realism and structural fidelity in the reconstruction of NeRF-based plants. These improvements are particularly valuable for downstream applications, such as tea bud counting and candidate picking-point estimation for harvesting- oriented perception, where precise structural representation and semantic separation are essential.

After training the neural radiation field model, we utilize the volume sampling module provided by Nerfstudio to reconstruct the scene in three dimensions and generate high-quality point cloud data that integrate geometric structure, appearance color, and semantic information. The model is designed with three cooperative components. The Density Field encodes the volume density of each spatial point, which enables effective separation of solid surfaces such as tea tree leaves and branches from the background. The Appearance Field associates each point with RGB color information to recover realistic lighting and material properties. The Semantic Field predicts the semantic probabilities of key targets such as tea buds, thereby supporting semantic-level 3D reconstruction.

**Figure 16:**
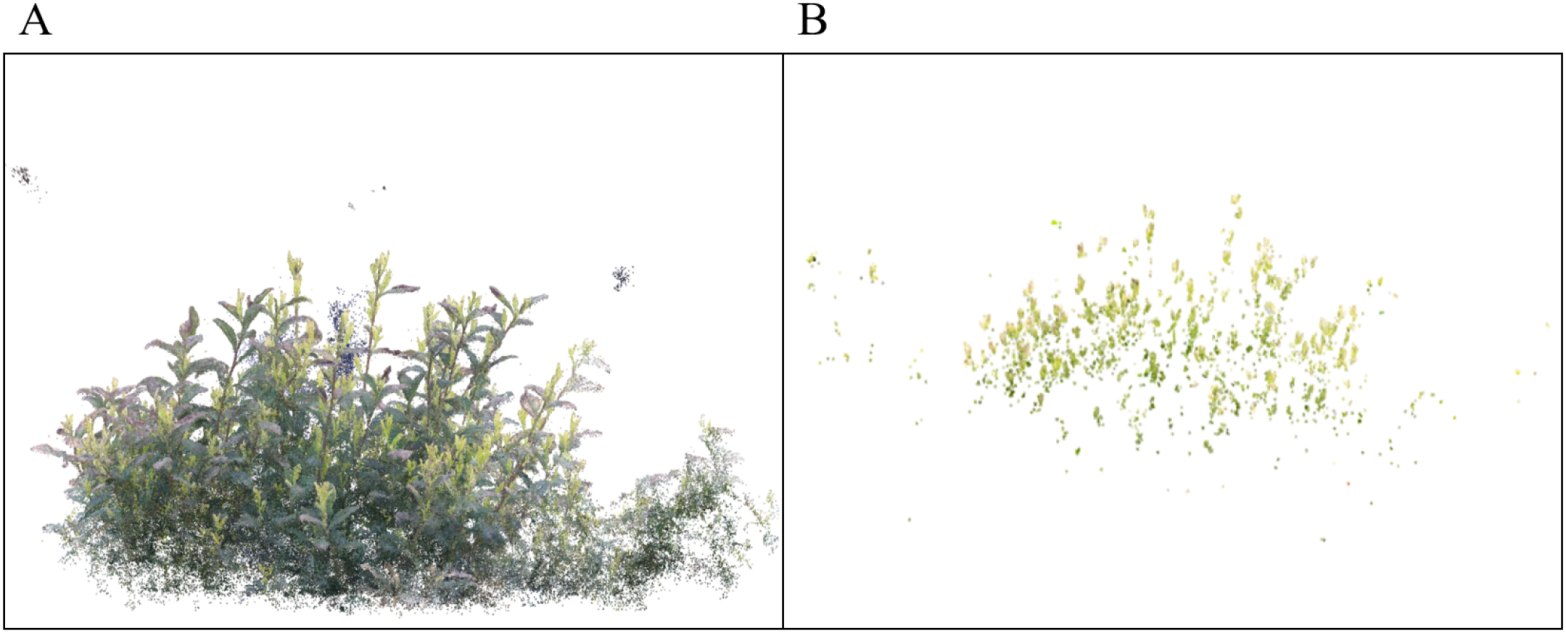
Tea tree and tea bud point clouds. **(A)** tea tree point cloud. **(B)** tea bud point cloud.

Nevertheless, during neural field training, semantic features may propagate along the line-of-sight direction, and direct sampling of the semantic field often introduces noisy labels in invalid regions, such as empty background space. To overcome this limitation, we propose a density-symmetric Coupling (DSC) strategy. In this method, a density threshold (*σ* ≥ 0.45) is applied and semantic predictions are only retained for spatial points where the density field indicates the presence of solid surfaces. This constraint effectively suppresses spurious semantic predictions in invalid space and ensures that semantic labels remain consistent with actual physical structures, as shown in **Figure 16**. By constraining semantic predictions with density responses, DSC suppresses a major source of structured semantic noise and improves the reliability of downstream clustering and counting.

Based on the reconstructed semantic point cloud, tea bud counting and harvesting-oriented candidate point estimation are performed as perception-level outputs to support downstream harvesting tasks. Once the point cloud data of the tea buds are obtained, a clustering algorithm is applied to structure the data for tea bud counting and candidate point estimation. Before clustering, the point clouds are preprocessed using Statistical Outlier Removal and Radius Outlier Removal to eliminate noise and anomalies, thereby improving the reliability of subsequent analysis. In practice, multiple small tea buds located in proximity may be geometrically merged into the same cluster, leading to biased counting results. To alleviate this issue, a DBSCAN-based clustering strategy with an adaptive neighborhood radius (guided by local point density) and a normal-consistency constraint (clamp angle ≤ 15^°^) is adopted, which helps reduce cluster adhesion in dense bud regions.

Following clustering, the RANSAC algorithm is applied to fit the dominant growth plane of each tea bud cluster. A distance threshold is then used to retain in-plane points, which are subsequently projected along the normal vector of the fitted plane. The point with the smallest projection value is selected as the harvesting-oriented candidate point for the corresponding tea bud cluster. It should be noted that this candidate point represents a perception-level three-dimensional guidance cue derived from geometric structure, rather than an anatomically exact stem–bud junction or a robot-executable cutting command.

**Figure 17:**
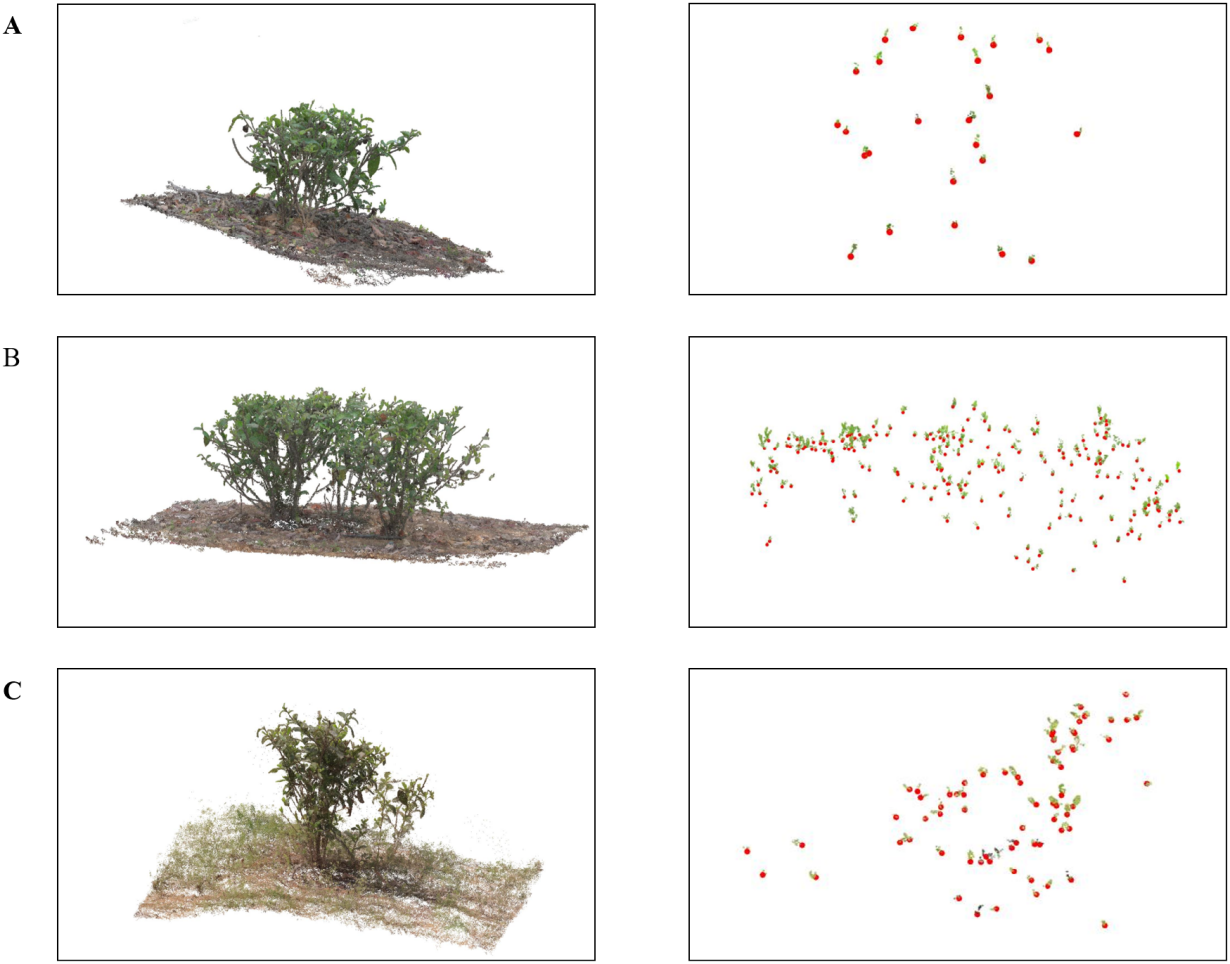
Illustration of tea bud counting and harvesting-oriented candidate point estimation. **(A–C)** Representative examples from three different tea tree scenes. For each row, the left panel shows the reconstructed tea tree point cloud, and the right panel shows the extracted tea bud point cloud. Red dots indicate harvesting-oriented candidate points.

To illustrate the feasibility of three-dimensional tea bud counting based on semantic point clouds under different occlusion conditions, three individual tea trees are selected as representative case studies. These trees are not randomly sampled; instead, they are deliberately chosen to reflect increasing levels of occlusion complexity, ranging from relatively sparse foliage to severe self-occlusion. The tea buds on each tree are manually harvested, and the corresponding counts are recorded as reference values. Three-dimensional reconstruction is then performed using NeRF, and the resulting semantic point clouds are used for clustering-based counting. The qualitative correspondence between reconstructed bud clusters and manually observed buds is visualized in **Figure 17**, while the case-level counting results are summarized in **Table 5**. This experiment is intended as an illustrative, case-level evaluation rather than a population-level statistical analysis.

**Table 5:** Case-level tea bud counting results for three representative tea trees with increasing occlusion complexity.

Tree	The number of predictions	GT
A	25	30
B	198	228
C	74	87

### 3.4 Proxy-based evaluation of candidate picking point localization

To quantitatively assess the localization reliability of candidate picking points under realistic conditions, we employ a proxy-based evaluation strategy that focuses on geometric stability under repeated perturbations. Specifically, for each tea bud cluster, the candidate picking point is re-estimated multiple times under random subsampling of the point cloud, and the variability of the resulting points is measured.

It should be clarified that this proxy-based evaluation does not aim to directly measure biological correctness with respect to manually annotated stem–bud junctions. Due to the lack of reliable junction-level ground truth for real tea plants at scale, the adopted evaluation focuses on repeatability and consistency, which are necessary properties for harvesting-oriented guidance cues used in downstream planning.

Three quantitative metrics are adopted to assess localization stability: (1) the mean point deviation *µ_p_*, which measures the average Euclidean distance between repeated estimations; (2) the 90th percentile deviation *p*90*_p_*, which reflects worst-case sensitivity under perturbation; and (3) the pass rate, defined as the proportion of estimations whose deviation falls below a predefined tolerance threshold. Lower values of *µ_p_* and *p*90*_p_*, together with a higher pass rate, indicate more stable and reliable candidate point estimation.

**Table 6** summarizes the proxy-based localization stability results across three individual tea trees under consistent experimental settings (*p* = 5, subsample ratio = 0.5, noise = 0). The proposed method (*ours*) achieves substantially lower spatial deviation than PCA-based baselines and demonstrates competitive stability compared with centroid-based approaches. Although the cluster centroid method yields the lowest numerical deviation and the highest pass rate, it tends to favor geometrically central locations that lack explicit harvesting-oriented structural cues, as it does not incorporate any basal-side or attachment-related priors of tea buds.

**Table 6:** Proxy-based localization stability evaluation of candidate picking point estimation methods across different tea trees (*p*  = 5, subsample ratio = 0.5, noise = 0).

Tree	Method	Localization stability metrics		
		$\mu_{p}↓$	$p90_{p}↓$	Pass rate ↑
Tree1	ours	0.00986	0.01583	0.0849
	low_centroid	0.01403	0.02396	0.0683
	ours_offset (dk=1.5)	0.01351	0.02129	0.0708
	ours_offset (dk=3.0)	0.02107	0.03306	0.0557
	ours_offset (dk=5.0)	0.03256	0.05051	0.0481
	cluster_centroid	0.00403	0.00645	0.6368
	pca_low	0.06240	0.08161	0.0038
Tree2	ours	0.01221	0.01976	0.0980
	low_centroid	0.01623	0.03545	0.0911
	ours_offset (dk=1.5)	0.01511	0.02438	0.0940
	ours_offset (dk=3.0)	0.02131	0.03443	0.0880
	ours_offset (dk=5.0)	0.03105	0.04957	0.0820
	cluster_centroid	0.00415	0.00636	0.4880
	pca_low	0.06877	0.08681	0.0000
Tree3	ours	0.01003	0.01646	0.2076
	low_centroid	0.01502	0.02545	0.1895
	ours_offset (dk=1.5)	0.01284	0.02050	0.1973
	ours_offset (dk=3.0)	0.01811	0.02913	0.1781
	ours_offset (dk=5.0)	0.02626	0.04258	0.1570
	cluster_centroid	0.00353	0.00560	0.6908
	pca_low	0.08069	0.10390	0.0027

To further analyze the sensitivity of the proposed method to candidate point displacement, an offset strategy is introduced, where the estimated candidate picking point is shifted along the negative normal direction of the fitted local plane by a scaled distance Δ*k*. As Δ*k* increases from 1.5 to 5.0, both *µ_p_* and *p*90*_p_* increase monotonically, accompanied by a gradual decrease in pass rate. This trend indicates that excessive displacement amplifies instability under perturbations, highlighting the importance of moderate offsets when incorporating harvesting-oriented geometric priors.

Overall, the results demonstrate that the proposed method provides a stable and task-consistent harvesting-oriented guidance cue under geometric perturbations. While centroid-based approaches achieve strong numerical stability, the proposed strategy integrates basal-side geometric priors that are more aligned with harvesting-oriented localization. Its localization objective focuses on harvesting-oriented geometric referencing rather than precise anatomical reconstruction of the stem–bud junction. The offset analysis further clarifies the trade-off between robustness and harvesting-oriented displacement in candidate point estimation.

## 4 Discussion

### 4.1 Multi-model fusion for tea bud semantic segmentation

The experimental results demonstrate that the proposed tea bud segmentation framework benefits from the complementary strengths of the large-scale foundation model SAM2 and the lightweight detector YOLOv11. Tea bud segmentation remains a challenging task due to limited annotated data and severe occlusion among buds and surrounding leaves, which often leads to boundary ambiguity and missed detections in complex canopy environments.

While SAM2 exhibits strong generalization capability on unseen samples, its performance on small-scale targets such as tea buds is constrained in the absence of effective localization cues. Conversely, the YOLOv11-based detector provides reliable coarse localization but may suffer from incomplete boundaries under heavy occlusion. By introducing YOLOv11 detection results as prompts for SAM2, the proposed fusion strategy reduces segmentation ambiguity for small and partially occluded buds, leading to improved segmentation consistency and reduced annotation effort.

Nevertheless, segmentation errors are not entirely eliminated. Residual over-segmentation and under-segmentation persist in regions with extreme occlusion or weak visual contrast, and such errors may propagate to subsequent 3D reconstruction stages. In particular, missed or truncated bud regions in 2D segmentation cannot be recovered in later processing, highlighting the critical role of segmentation recall in harvesting-oriented 3D perception pipelines.

### 4.2 3D reconstruction effect

Most existing tea bud studies focus on recognition and localization in two-dimensional images, where occlusion and perspective effects fundamentally limit reliable structural analysis. By introducing a NeRF-based reconstruction framework with monocular depth priors, this study extends tea bud perception into three-dimensional space, enabling bud counting and candidate picking-point estimation from a geometric perspective.

Incorporating depth information derived from Depth Anything v2 into the Nerfacto framework improves reconstruction sharpness and structural fidelity, particularly during the early stages of training and in regions with complex leaf–bud interactions. From a methodological perspective, NeRF-based reconstruction provides a more flexible representation for modeling fine-scale plant organs with severe self-occlusion and repetitive textures, which often pose challenges to traditional structure-from-motion pipelines (e.g., COLMAP) by degrading feature matching and geometric consistency.

Nevertheless, reconstruction uncertainty remains an inherent challenge when modeling tea trees with fine-grained geometry and heavy self-occlusion. Monocular depth estimation is prone to ambiguity around thin structures and densely layered foliage, leading to local geometric blur or incomplete surfaces in the reconstructed space. Such errors are spatially correlated rather than random and may propagate into the semantic field and subsequent point cloud extraction, although depth supervision substantially suppresses reconstruction noise.

### 4.3 Depth error analysis in ambiguous regions

While monocular depth supervision improves the overall geometric consistency of NeRF reconstruction, it is inherently subject to estimation errors in visually ambiguous regions such as thin branches and dense foliage. To quantitatively assess how such depth errors may affect the reconstructed geometry, we conduct a region-aware depth reliability analysis by comparing Depth Anything V2 predictions with sparse depth estimates derived from COLMAP reconstruction.

Specifically, sparse 3D points reconstructed by COLMAP are projected onto the image plane, and their camera-space depths are treated as reference depth samples. At corresponding pixel locations, monocular depth predictions are extracted and aligned using robust median scaling to account for the inherent scale ambiguity of monocular depth estimation. Depth errors are then evaluated for all valid samples (Global), manually selected ambiguous regions of interest (ROIs), including thin branches and dense foliage, and non-ROI regions as a control group.

Quantitative results are summarized in **Table 7**. The global region exhibits moderate depth errors, with performance comparable to that of non-ROI regions, indicating that monocular depth supervision provides reasonably consistent structural guidance at the scene level. In contrast, error magnitudes increase noticeably in visually ambiguous regions. Thin-branch ROIs show moderately higher errors, reflecting the difficulty of estimating depth for fine, elongated structures. Dense-foliage ROIs exhibit substantially larger absolute and relative errors, together with significantly heavier-tailed error distributions, as evidenced by the elevated p90 AbsRel values. This behavior can be attributed to severe occlusion, multi-layer leaf overlap, and weak monocular depth cues in dense foliage areas.

**Table 7:** ROI-based depth error statistics after scale alignment.

Region	Points	AbsRel ↓	RMSE ↓	p90 AbsRel ↓
Global	24,765	1.507	9.727	3.975
Thin branches ROI	402	1.826	10.862	3.243
Dense foliage ROI	505	4.234	15.008	7.487
Non-ROI	23,858	1.444	9.563	3.859

These results indicate that depth estimation errors are not uniformly distributed across the scene, but are spatially concentrated in regions with inherent visual ambiguity. Consequently, monocular depth supervision may introduce localized geometric blur or noise in such regions, while preserving global structural fidelity. This region-dependent error characteristic explains the localized reconstruction artifacts observed in dense foliage areas and supports the subsequent stability analysis of candidate picking point estimation.

### 4.4 Analysis of tea bud counts and harvesting-oriented candidate points

Most existing tea bud yield estimation approaches rely on two-dimensional images, where occlusion and projection overlap frequently lead to counting ambiguity. By leveraging a reconstructed semantic point cloud, the proposed method enables tea bud counting from a three-dimensional perspective, which helps alleviate ambiguity caused by overlapping buds in 2D views. Rather than serving as a population-level statistical evaluation, the counting results presented in this study are intended as a case-level illustration to examine the feasibility and behavior of 3D-based counting under different occlusion conditions.

As observed in the representative cases, discrepancies between reconstructed counts and manual reference values still occur. Under-counting typically arises when severely occluded tea buds are incompletely reconstructed and fail to form sufficiently dense point cloud clusters, or when closely adjacent buds are geometrically merged into a single cluster. In contrast, partial fragmentation of an individual bud may occasionally lead to over-counting. These error patterns are not randomly distributed; instead, they reflect the stage-wise accumulation of uncertainty across segmentation, depth estimation, and point cloud clustering, rather than isolated inaccuracies at a single processing step.

For harvesting-oriented candidate point estimation, existing approaches predominantly rely on two-dimensional localization cues or point clouds acquired through LiDAR scanning. In contrast, this study derives candidate points directly from reconstructed semantic point clouds by combining bud-level clustering with RANSAC-based local geometric modeling. The resulting candidate points represent perception-level three-dimensional guidance cues that capture the relative spatial structure of tea buds, rather than anatomically exact stem–bud junctions. Although their spatial consistency depends on the completeness of bud reconstruction and the stability of cluster-level geometric fitting, these candidate points provide actionable spatial references for downstream harvesting planning. Systematic validation across larger-scale datasets and explicit occlusion-level stratification are left for future work.

## 5 Limitations

Despite the promising results achieved by the proposed pipeline, several limitations remain. First, the proposed framework is designed for high-precision, single-tree-level three-dimensional perception and harvesting planning, where reconstruction quality and geometric reliability are prioritized over processing throughput. Consequently, the current implementation targets offline analysis and is not intended for real-time deployment in large-scale tea gardens, where efficiency and coverage are critical considerations.

Second, the performance of the system is sensitive to data acquisition conditions. Reliable reconstruction requires relatively stable environmental settings, including limited wind disturbance and reasonably uniform illumination. Under dynamic outdoor conditions with strong wind or rapidly changing lighting, image quality degradation may affect both reconstruction accuracy and downstream point cloud analysis.

Third, the dataset used in this study was collected from a single geographic region and a single tea variety, which limits a comprehensive evaluation of cross-region and cross-variety generalization. However, the primary objective of this work is to validate a harvesting-oriented three-dimensional perception framework rather than to claim universal generalization across diverse tea species or plantation conditions. The proposed pipeline relies on general visual and geometric cues, such as object appearance, depth structure, and spatial consistency, rather than handcrafted features specific to a particular tea variety. Systematic evaluation on multi-region and multi-variety datasets will be explored in future work.

In addition, the evaluation of picking-point estimation remains challenging. Due to the dense distribution and large quantity of tea buds on a single tea tree, it is difficult to obtain precise ground-truth picking locations for direct quantitative comparison. Consequently, the current validation primarily focuses on perceptual consistency rather than execution-level accuracy.

Addressing these limitations will require further investigation, including improving data acquisition robustness under complex environmental conditions, enhancing system efficiency for larger-scale scenarios, expanding dataset diversity, and developing reliable strategies for validating picking-point estimates under practical harvesting constraints.

## 6 Conclusions

This study presents a harvesting-oriented three-dimensional perception framework for tea bud analysis that integrates two-dimensional recognition and segmentation with depth-assisted NeRF reconstruction and semantic point cloud processing. By combining lightweight detection, prompt-guided semantic segmentation based on SAM2, and monocular depth priors, the proposed pipeline improves robustness under occlusion and enables structured three-dimensional representation of tea buds in complex plantation environments.

Based on the reconstructed semantic point cloud, the framework provides two perception-level outputs relevant to harvesting applications: tea bud counting and harvesting-oriented candidate point estimation. Experimental results on captured tea tree scenes indicate that the estimated tea bud counts are generally consistent with manual measurements, demonstrating the potential of three-dimensional perception to support yield estimation. In addition, the estimated candidate points capture the relative spatial structure of tea buds and serve as three-dimensional spatial references that can support downstream harvesting planning.

Overall, this work demonstrates the feasibility of unifying two-dimensional visual perception and neural field-based 3D reconstruction into a single pipeline for harvesting-oriented tea bud analysis. While further improvements are required to enhance scalability, robustness under varying environmental conditions, and execution-level validation, the proposed framework establishes a solid perceptual foundation for future research on intelligent tea harvesting systems. 

## Data Availability

The raw data supporting the conclusions of this article will be made available by the authors, without undue reservation.
