# Supplementary material for: TeaNeRF: an integrated 3D visual perception pipeline for tea bud harvesting
Source: Front Plant Sci. 2026 Mar 2;17:1739203. doi: 10.3389/fpls.2026.1739203 (PMC12989544; doi:10.3389/fpls.2026.1739203)
Supplement: Supplementary file 1 [file DataSheet1.pdf]

Supplementary Material

1 SUPPLEMENTARY DATA

1.1 Table S1. Key hyperparameters critical to reproducibility

Module	Hyperparameter	Value	Description
DBSCAN	eps	0.01	Neighborhood radius for density-based clustering
DBSCAN	minPts	30	Minimum number of points to form a core point
DBSCAN	min_cluster_points	50	Minimum cluster size retained for further processing
Semantic filtering	σ	≥ 0.45	Density confidence threshold
NeRF training	λ_{sem}	1.0	Semantic loss weight
NeRF training	λ_{depth}	0.1	Depth loss weight
RANSAC	τ_{inlier}	$2.5 \times d_{nn}$	Inlier threshold
RANSAC	iterations	300	Number of RANSAC iterations

d_{nn} denotes the average nearest-neighbor distance of the point cloud. All hyperparameters are fixed across experiments unless otherwise stated.

1.2 Table S2. Approximate runtime breakdown of the TeaNeRF pipeline

Pipeline stage	Typical runtime
Data acquisition	∼30 s
Data preprocessing	∼15 min
2D image segmentation	∼2 min
3D reconstruction	∼5 min
Point cloud processing	∼4 min

1.3 Figure S1. Qualitative comparison between NeRF and COLMAP dense reconstruction

This figure presents a qualitative comparison between a NeRF-based reconstruction and a traditional structure-from-motion and multi-view stereo (SfM-MVS) approach, namely COLMAP dense reconstruction, for the same tea plant scene. The purpose of this comparison is to provide an illustrative analysis of typical reconstruction behavior under fine-scale plant structures with repetitive textures and self-occlusion. This comparison is qualitative in nature and is not intended to serve as a quantitative benchmark or performance evaluation.

Both reconstructions are generated from the same set of input images after identical image screening based on lighting conditions and sharpness, following the preprocessing procedure described in Section 2.3 of the main paper. Camera poses are estimated using COLMAP for both methods. The NeRF-based reconstruction adopts vanilla Nerfacto as the baseline, while COLMAP dense reconstruction is performed using standard settings.

As shown in Figure S1, COLMAP dense reconstruction tends to produce sparse and noisy point clouds, with fragmented or incomplete structures in regions corresponding to thin branches and dense foliage. Such artifacts are commonly observed in plant scenes characterized by repetitive textures and frequent self-occlusion, where feature matching and triangulation become unstable. In contrast, the NeRF-based reconstruction yields a more spatially coherent point cloud, preserving the overall plant structure and

providing denser geometric support for subsequent harvesting-oriented semantic point cloud processing. This figure is provided for illustrative purposes only.

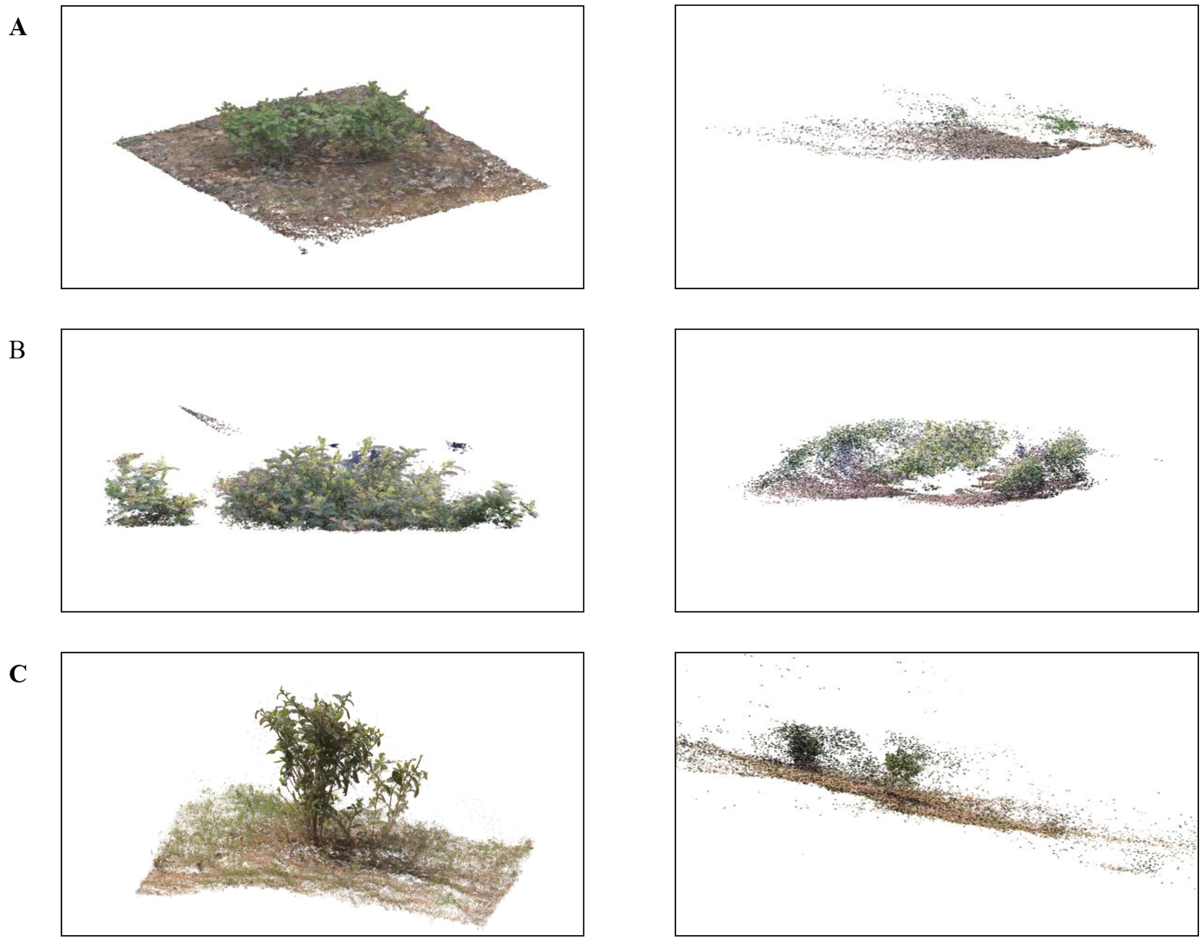

Figure S1. Qualitative comparison of point clouds reconstructed using NeRF and COLMAP dense reconstruction. The left column shows the NeRF-based reconstruction, while the right column shows the COLMAP dense reconstruction. Each row presents the same tea plant scene viewed from different viewpoints. Differences in point cloud density and structural continuity can be observed, particularly in regions with thin branches, dense foliage, and ground surfaces. This figure is provided for illustrative purposes only.
